# DNA damage response defects induced by the formation of TDP-43 and mutant FUS cytoplasmic inclusions and their pharmacological rescue

**DOI:** 10.1038/s41418-025-01530-7

**Published:** 2025-05-29

**Authors:** Stefania Modafferi, Stefania Farina, Francesca Esposito, Ornella Brandi, Michela Di Salvio, Ilaria Della Valle, Sara D’Uva, Eveljn Scarian, Giada Cicio, Adelaide Riccardi, Federica Pisati, Anna Garbelli, Tiziana Santini, Orietta Pansarasa, Mariangela Morlando, Nadia D’Ambrosi, Mauro Cozzolino, Gianluca Cestra, Fabrizio d’Adda di Fagagna, Ubaldo Gioia, Sofia Francia

**Affiliations:** 1 Institute of Molecular Genetics “Luigi Luca Cavalli-Sforza”, National Research Council (CNR), Pavia, Italy; 2 Institute of Molecular Biology and Pathology, CNR, Rome, Italy; 3 PhD Program in Cellular and Molecular Biology, Department of Biology, University of Rome Tor Vergata, Rome, Italy; 4 Department of Biology and Biotechnologies “Charles Darwin”, Sapienza University of Rome, Rome, Italy; 5 Cellular Models and Neuroepigenetics Unit, IRCCS Mondino Foundation, Pavia, Italy; 6 IFOM ETS—The AIRC Institute of Molecular Oncology, Milan, Italy; 7 Cogentech Società Benefit srl, Milan, Italy; 8 Department of Biology, University of Rome Tor Vergata, Rome, Italy; 9 Institute of Translational Pharmacology, CNR, Rome, Italy; 10 Fondazione Policlinico Universitario A. Gemelli IRCCS, Università Cattolica del Sacro Cuore, Rome, Italy; 11 Present Address: Department of Life, Health and Environmental Sciences, University of L’Aquila, L’Aquila, Italy

**Keywords:** Molecular biology, Cell biology

## Abstract

Formation of cytoplasmic inclusions (CIs) of TDP-43 and FUS, along with DNA damage accumulation, is a hallmark of affected motor neurons in Amyotrophic Lateral Sclerosis (ALS). However, the impact of CIs on DNA damage response (DDR) and repair in this pathology remains unprobed. Here, we show that CIs of TDP-43 and FUS^P525L^, co-localizing with stress granules, lead to a dysfunctional DDR activation associated with physical DNA breakage. Inhibition of the activity of the DDR kinase ATM, but not of ATR, abolishes DDR signaling, indicating that DNA double-strand breaks (DSBs) are the primary source of DDR activation. In addition, cells with TDP-43 and FUS^P525L^ CIs exhibit reduced DNA damage-induced RNA synthesis at DSBs. We previously showed that the two endoribonucleases DROSHA and DICER, also known to interact with TDP-43 and FUS during small RNA processing, contribute to DDR signaling at DSBs. Treatment with enoxacin, which stimulates DDR and repair by boosting the enzymatic activity of DICER, restores a proficient DDR and reduces DNA damage accumulation in cultured cells with CIs and in vivo in a murine model of ALS. In *Drosophila melanogaster*, Dicer-2 overexpression rescues TDP-43-mediated retinal degeneration. In summary, our results indicate that the harmful effects caused by TDP-43 and FUS CIs include genotoxic stress and that the pharmacological stimulation of the DNA damage signaling and repair counteracts it.

## Introduction

Amyotrophic Lateral Sclerosis (ALS) is a neurodegenerative disease characterized by the progressive loss of the upper and lower motor neurons due to neuronal cell death [1]. Analogously to other neurodegenerative disorders like Frontotemporal Dementia (FTD) and Alzheimer’s disease (AD), ALS is defined as a proteinopathy since motor neurons of patients affected by this pathology harbor aberrant protein aggregates in their cytoplasm [2]. 90% of ALS cases are sporadic (sALS), thus lacking a familial history, while the remaining ALS cases are characterized by autosomal dominant, autosomal recessive, or X-linked mutations occurring in specific genes, leading to familial inheritance (familial ALS, fALS) [3]. Notably, around 3% of fALS-associated mutations fall in the gene encoding for TAR DNA-binding protein 43 (TDP-43) and 6% in the gene for fused in sarcoma (FUS) and often follow an autosomal dominant inheritance pattern [3, 4]. Some mutations, particularly FUS^P525L^, are believed to confer to these proteins an increased predisposition to form cytosolic aggregates [5]. Nevertheless, in the vast majority of both sALS and fALS and in FTD cases, TDP-43 accumulates in cytoplasmic inclusions (CIs) in its wild-type form [6]. In vivo, expression of mutant or wild-type human TDP-43 causes reduced locomotor activity and progressive paralysis in mice, and decreased lifespan in fruit flies [4]. FUS mutations also lead to a range of phenotypes affecting the nervous system, including defects in neuronal development, locomotion problems, reduced survival, and neurodegeneration in various model organisms [7]. FUS and TDP-43 knockout mice (FUS^−/−^ and TARDBP^−/−^, respectively) are often perinatal lethal while heterozygous animals exhibit motor symptoms [7, 8]. FUS^−/−^ mice also show male sterility, defects in B-lymphocyte development, increased genomic instability and vulnerability to ionizing radiation [7].

TDP-43 and FUS are two RNA-binding proteins (RBPs) that cover multiple functions in RNA metabolism. They are both involved in microRNA (miRNA) biogenesis, through their interaction with the endoribonuclease DROSHA [9, 10]. Moreover, FUS also exhibits a transcriptional regulatory role and can directly bind to the C-terminal domain (CTD) of RNA polymerase II (RNAPII) [11, 12]. Both TDP-43 and FUS are structurally characterized by the presence of low complexity domains (LCDs), which allow the two proteins to undergo liquid-liquid phase separation (LLPS), essential for the formation of insoluble membrane-less organelles, including stress granules (SGs) [13].

SGs are generated in response to different stress sources, with the proposed function of pausing protein synthesis until the stress ends [14]. Along with their constitutive components, like TIA-1 and G3BP1, SGs may include TDP-43 and FUS [14]. Interestingly, TDP-43 depletion alters both G3BP1 and TIA-1 levels and affects SG structure and dynamics [15]. Besides, motor neurons derived from induced pluripotent stem cells (iPSCs) carrying the FUS^P525L^ mutation display an increased number and altered kinetics of SG assembly [16].

Genome integrity ensures the maintenance of the genetic information against possible unfaithful transmission across generations. Both physiological events and environmental factors can cause DNA breaks [17]. Therefore, cells have developed a complex pathway named DNA Damage Response (DDR), which immediately detects the DNA lesion, signals its formation and ensures a proper DNA repair [18]. In particular, following double-strand break (DSB) formation, the multiprotein complex MRN (MRE11-RAD50-NBS1) binds the DNA ends, thus recruiting and activating ATM, a member of the phosphatidylinositol 3-kinase-related kinase (PIKK) family [19]. ATM, in turn, initiates the DDR signaling by phosphorylating the histone variant H2AX, which in its phosphorylated version is referred to as γH2AX [20]. Upon accumulation of single-strand breaks (SSBs), generally occurring during replication stress, H2AX is phosphorylated by ATR, another PIKK [21]. Initial γH2AX chromatin decoration creates a positive feedback loop that fuels further spreading of γH2AX and sequential accumulation of DDR proteins [20], including 53BP1, which plays a pivotal role in orchestrating the choice of DSB repair pathways [22].

Different neurodegenerative diseases, including ALS, have been associated with a high accumulation of DNA lesions in the nucleus of motorneurons [23, 24], affecting their viability. Intriguingly, a role for TDP-43 and FUS in DNA repair has been recently identified in loss of function model systems [25, 26]. For instance, the knockdown of either FUS or TDP-43 has been found to generate DNA breaks via RNAPII transcription stalling and the formation of DNA:RNA hybrids [27, 28]. Additionally, TDP-43 depletion hampers the recruitment to DSBs of the XRCC4-DNA ligase IV complex, which is crucial for the non-homologous end-joining (NHEJ) DNA repair pathway [25]. Similarly, FUS loss reduces the association of the repair protein Ku70 to DSB sites, and the recruitment of XRCC1/LIG3 to SSBs, resulting in the accumulation of unrepaired DNA lesions [25]. Instead, the impact of the formation of TDP-43- and FUS-containing SGs on genome integrity and DDR activation remains unknown.

In the present study, we investigate the molecular mechanisms underlying defects in DDR activation and the consequent accumulation of DNA damage following the formation of TDP-43 and FUS CIs. We also provide evidence that demonstrate that DDR components are potential therapeutic targets to mitigate the neurotoxic effects of TDP-43 and FUS proteinopathies.

## Results

### Formation of TDP-43 and mutant FUS cytoplasmic inclusions (CIs) is associated with DNA damage accumulation

To acutely induce the formation of CIs in human cultured cells, we transiently transfected HeLa cells with plasmids expressing wild-type (WT) FUS and its ALS-linked mutant form (FUS^P525L^), along with WT TDP-43 and two ALS-related mutant derivatives (TDP-43^A382T^ and TDP-43^I383V^), in parallel with an empty vector (EV) as a control. Western blot analyses confirmed that the amounts of the ectopically expressed wild-type and mutant TDP-43 and FUS proteins were comparable to their endogenous levels (Fig. S1A, B). We next probed for FUS and TDP-43 subcellular localization by immunofluorescence (IF), using antibodies against the endogenous proteins. Although both FUS and TDP-43 were predominantly nucleoplasmic, they segregated into CIs (bright cytoplasmic puncta positive for FUS or TDP-43) in approximately 30% and 15–20% of the cells overexpressing FUS^P525L^ and TDP-43, respectively (Fig. S1C–F). Differently, the fraction of cells showing endogenous TDP-43-positive CIs was much lower (less than 3%) in samples transfected with the EV (Fig. S1C, E). Importantly, FUS segregated to CIs only upon the expression of its mutant version, while it was retained in the nucleus in WT FUS-expressing cells (Fig. S1D, F). Differently, ALS-linked mutations of TDP-43 did not further increase the tendency of this protein to form CIs (Fig. S1C, E). This observation aligns with the prevalence of WT TDP-43 inclusions in most ALS patients [6]. Consistently with previous reports [29], the expression of FUS^P525L^ and TDP-43 induced the formation of SGs, as determined by IF through the co-staining for the SG markers TIA-1 and G3BP1 (Fig. S1G). Importantly, the majority of FUS^P525L^ and TDP-43 CIs colocalized with such organelles (Fig. S1G, H), as also observed in ALS brain tissues [29]. Since the formation of CIs upon expression of either WT or mutant TDP-43 did not exhibit significant differences (Fig. S1C, E), and given our research focus on investigating the impact of CI formation on DDR activation and DNA repair, we exclusively conducted experiments using cells expressing WT TDP-43 for the rest of this study.

**Fig. 1:**
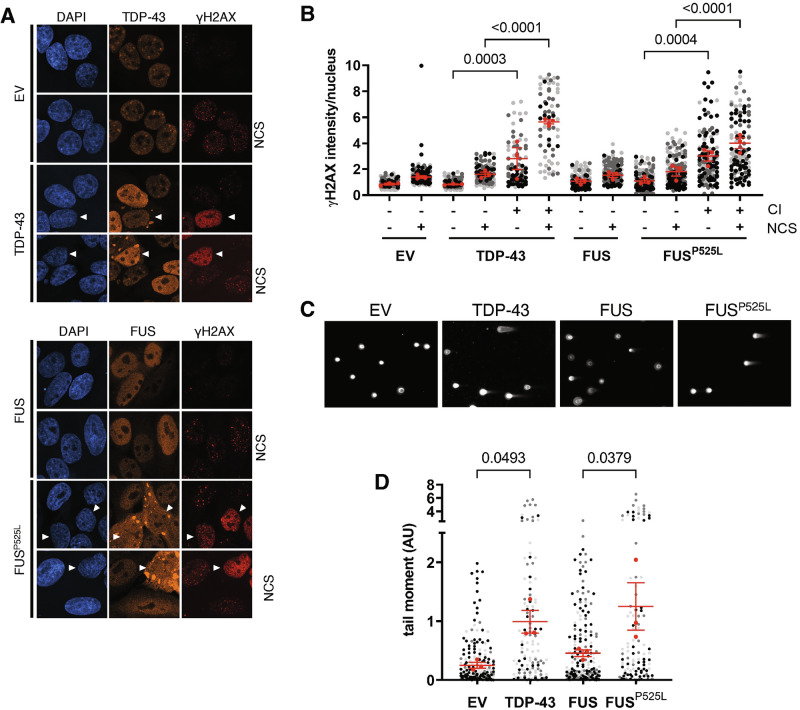
Cells with TDP-43 or FUS^P525L^ cytoplasmic inclusions (CIs) show elevated γH2AX levels and increased DSB formation. **A** Analysis by immunofluorescence (IF) of γH2AX signals (red) in damaged (NCS) or undamaged HeLa cells transfected with plasmids expressing TDP-43, FUS or FUS^P525L^. Cells transfected with an empty vector (EV) were used as a control. TDP-43 and FUS signals are shown in orange; nuclei were counter-stained with DAPI (blue). Arrowheads mark cells with CIs. **B** Super-plot showing the quantification of γH2AX intensity in HeLa cells with or without cytoplasmic inclusions (± CI) determined in (**A**). Red dots represent the mean values of each biological replicate; red bars indicate the averages ± SEM of three independent experiments. **C** Representative images of comet assay experiments performed in undamaged HeLa cells transfected as in (**A**). **D** Tail moment analysis of HeLa cells shown in (**C**). The red dots of the super-plot represent the mean values of each biological replicate; red bars indicate the averages ± SEM of three independent experiments.

We then investigated the effects of CI formation on DNA damage accumulation at single-cell resolution by treating FUS- or TDP-43-expressing cells with Neocarzinostatin (NCS)—a radiomimetic drug largely used to acutely generate DSBs [30, 31]—followed by IF analysis. Notably, localization of FUS^P525L^ or TDP-43 into CIs was not affected by NCS administration (Fig. S1C–F), indicating that DNA damage is not the cause of FUS^P525L^ or TDP-43 aggregation, but rather a consequence. WT FUS- or EV-transfected cells presented little endogenous DNA damage and properly formed γH2AX-positive DDR foci upon treatment with NCS (Fig. 1A, B). Differently, a robust accumulation of pan-nuclear γH2AX signal was observed in cells with mutant FUS and TDP-43 CIs (Fig. 1A, B). Such a massive amount of γH2AX occurred even in the absence of exogenously inflicted DNA damage (Fig. 1A, B), indicating that the formation of CIs following FUS^P525L^ or TDP-43 expression was sufficient to induce a significant genotoxic stress. This intense γH2AX signal prevents from distinguishing discrete γH2AX foci in NCS-treated cells (Fig. 1A, B). Importantly, adjacent cells without mutant FUS- or TDP-43 CIs did not display pan-nuclear γH2AX signal, both in undamaged and damaged conditions (Fig. 1A, B). Augmented γH2AX levels are indicative of an increase of unrepaired DSBs. Thus, to specifically detect DSB accumulation following FUS or TDP-43 expression, we performed a neutral comet assay in cells not exposed to exogenous DNA damage and transfected with EV, FUS, FUS^P525L^ or TDP-43. Interestingly, we observed that the expression of TDP-43 or FUS^P525L^ increased the comet tail moment, suggestive of a higher amount of DSBs, compared to EV-transfected samples or cells overexpressing WT FUS (Fig. 1C, D).

These results indicate that the elevated γH2AX signal observed in the nucleus of cells with mutant FUS- or TDP-43- CIs is associated with a significant increase of physical DNA breakage in the form of DSBs.

### ATM is hyper-activated and responsible for the increased γH2AX levels in cells with CIs

**Fig. 2:**
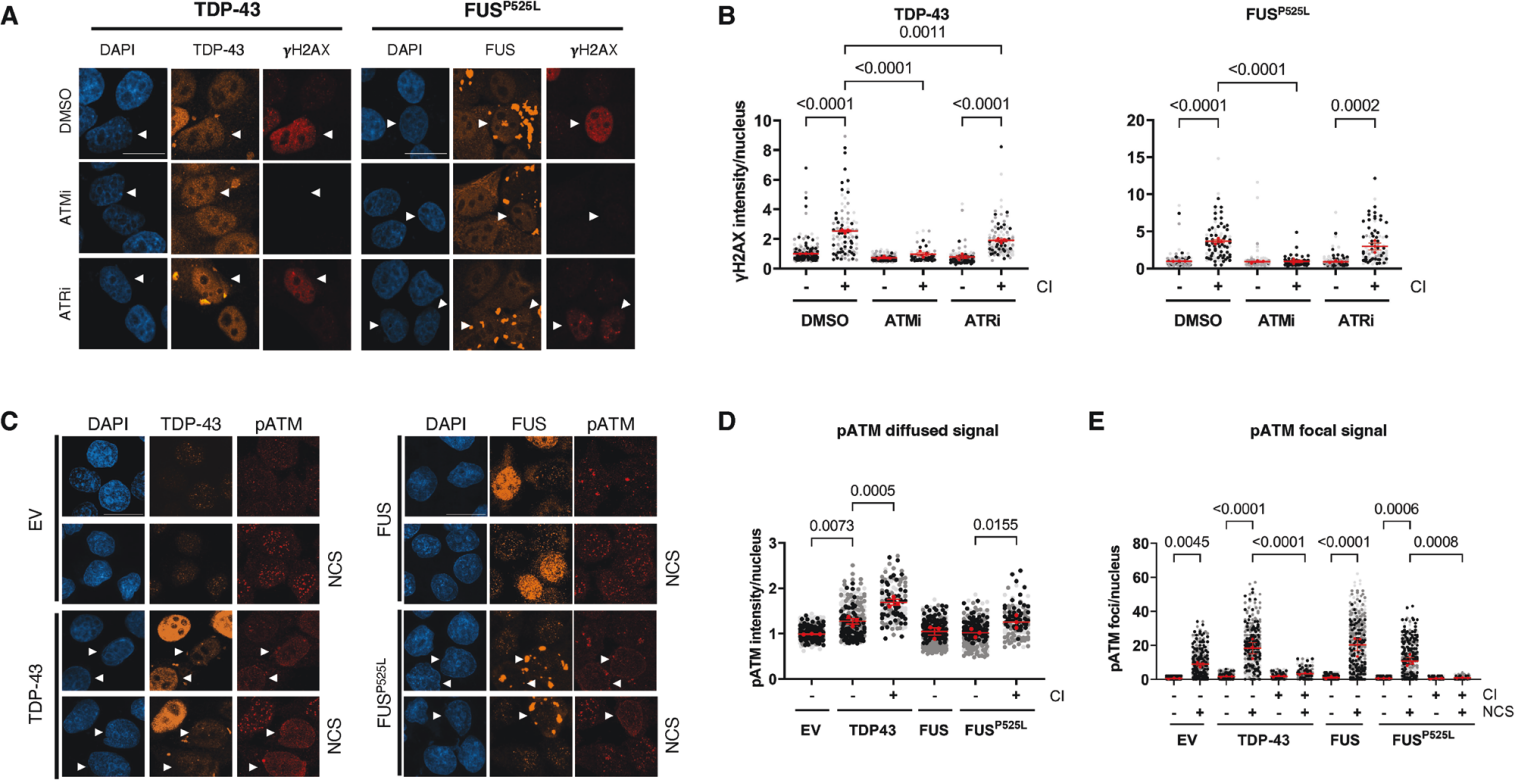
ATM is aberrantly activated, and its inhibition abolishes pan-nuclear γH2AX formation in cells with TDP-43 and FUS^P525L^ CIs. **A** Analysis by immunofluorescence (IF) of γH2AX levels (red) in HeLa cells expressing TDP-43 or FUS^P525L^ and treated with ATMi or ATRi. Cells treated with DMSO were used as a control. TDP-43 and FUS are labeled in orange; nuclei were counter-stained with DAPI. Arrowheads mark cells with CIs; scale bar = 10 µm. **B** Quantification of γH2AX intensity in HeLa cells with or without cytoplasmic inclusions (±CI) described in (**A**). The red dots of the super-plots represent the mean values of each biological replicate; red bars indicate the averages ± SEM of three independent experiments. **C** IF analysis of pATM signal (red) in HeLa cells transfected with plasmids expressing an empty vector (EV), TDP-43 or FUS^P525L^. TDP-43 and FUS are labeled in orange; nuclei were counter-stained with DAPI. Arrowheads mark cells with CIs; scale bar = 10 µm. **D**, **E** Quantification of diffused and focal pATM signal (**D**, **E**, respectively) in HeLa cells with or without cytoplasmic inclusions (± CI) determined in (**C**). The red dots of the super-plot represent the mean values of each biological replicate; red bars indicate the averages ± SEM of three independent experiments.

As soon as the DNA damage is sensed, the apical kinases ATM and ATR are engaged and trigger the local phosphorylation of H2AX, along with several other protein targets [20, 21]. A previous study showed that pan-nuclear γH2AX accumulates in response to ATM activation in different contexts of chromatin structure alteration [32]. Instead, ATR activation has been occasionally associated with γH2AX pan-nuclear signal during replication stress [33, 34]. Thus, to determine which of these two kinases was responsible for γH2AX accumulation observed, we treated cells expressing FUS^P525L^, TDP-43, or control samples, with specific ATM or ATR kinase activity inhibitors (KU60019 or VE-821, respectively [35]) and monitored their impact on γH2AX signals by IF. The specificity of inhibition of the two DDR kinases was confirmed by monitoring their auto-phosphorylation through immunoblotting (Fig. S2A). Intriguingly, ATM inhibition almost completely abrogated γH2AX signal in cells with TDP-43 or FUS^P525L^ CIs, compared to control samples treated with the vehicle (DMSO) (Fig. 2A, B). On the other hand, ATR inactivation had no effect in decreasing γH2AX levels in cells with FUS^P525L^, while it marginally reduced γH2AX signal in those with TDP-43 CIs (Fig. 2A, B).

We also observed that cells not treated with NCS and harboring TDP-43 or FUS^P525L^ CIs exhibited an aberrantly diffused pATM signal, in contrast to those expressing TDP-43 or FUS^P525L^ but lacking CIs (Fig. 2C, D). This suggests that the formation of such aggregates is sufficient to trigger a diffused and aberrant ATM activation even without exogenous induction of DNA damage. In line with this, we also observed that ATM activation is inefficient in cells with CIs. While cells without FUS^P525L^ or TDP-43 CIs properly mounted pATM foci formation upon DSB induction following NCS treatment, cells harboring CIs showed a significantly lower number of pATM foci (Fig. 2C, E). When we next probed by IF for the activation by phosphorylation of the transducer kinase CHK2, an ATM substrate [36], CI-bearing cells displayed reduced pCHK2 signals upon NCS treatment (Fig. S2B, C).

In sum, these results indicate that the accumulation of pan-nuclear γH2AX detected in CI-bearing cells relies on a dysfunctional ATM activation, which is responsible for pan-nuclear γH2AX levels but does not efficiently transmit the DDR signal to downstream effector kinases, such as CHK2.

Previous evidence indicates that pan-nuclear γH2AX marks cells in S-phase upon UV-irradiation [37] and correlates with DNA replication defects [34]. Intriguingly, TDP-43 was shown to associate with replication forks in normal conditions, protecting cells from replicative stress [38]. Although we observed that ATR, involved in regulating DDR during DNA replication, was dispensable for γH2AX accumulation (Fig. 2A, B), we tested if the γH2AX signal observed in cells with FUS^P525L^ or TDP-43 CIs might be elicited by replicative stress. To this end, we initially examined the capacity of CI-bearing cells to de novo synthesize DNA, by performing a 5-bromo-2′-deoxyuridine (BrdU) incorporation assay followed by IF. We observed that cells with FUS^P525L^ or TDP-43 CIs incorporated much less BrdU, compared to those without CIs or with samples expressing WT FUS or the EV (Fig. S2D, E). Similarly, we observed that all the cells harboring FUS^P525L^- or TDP-43-positive CIs had very low or null levels of Cyclin A (Fig. S2F), which is expressed in the S/G2-phase of the cell cycle [39, 40], suggesting that such cells were preferentially in G1-phase and not replicating. To confirm this, we took advantage of Fluorescent, Ubiquitination-based Cell Cycle Indicator (FUCCI)-expressing HeLa cells [41], which allow cell cycle phases to be readily visualized by fluorescent microscopy. These cells were transfected with plasmids expressing either TDP-43, FUS or FUS^P525L^, or with an empty vector (EV) as a control. We observed that the vast majority of cells presenting TDP-43 and FUS^P525L^ CIs (96 and 91%, respectively) were G1-positive (i.e., expressing the G1 marker CDT1, labeled in orange; Fig. S2G, H). In contrast, only 60–70% of cells without CIs or those expressing wild-type FUS or EV were G1-positive (Fig. S2G, H). These results, which are in line with those obtained through BrdU and Cyclin A staining, further support the impact of CI formation on impeding G1-S transition.

In summary, we found that CI formation causes DNA damage accumulation and an aberrant ATM activation, which in turn prevent cells from initiating DNA synthesis and entering the S-phase of the cell cycle.

### FUS^P525L^ and TDP-43 CIs hamper the phosphorylation and the recruitment of 53BP1 to DSBs

**Fig. 3:**
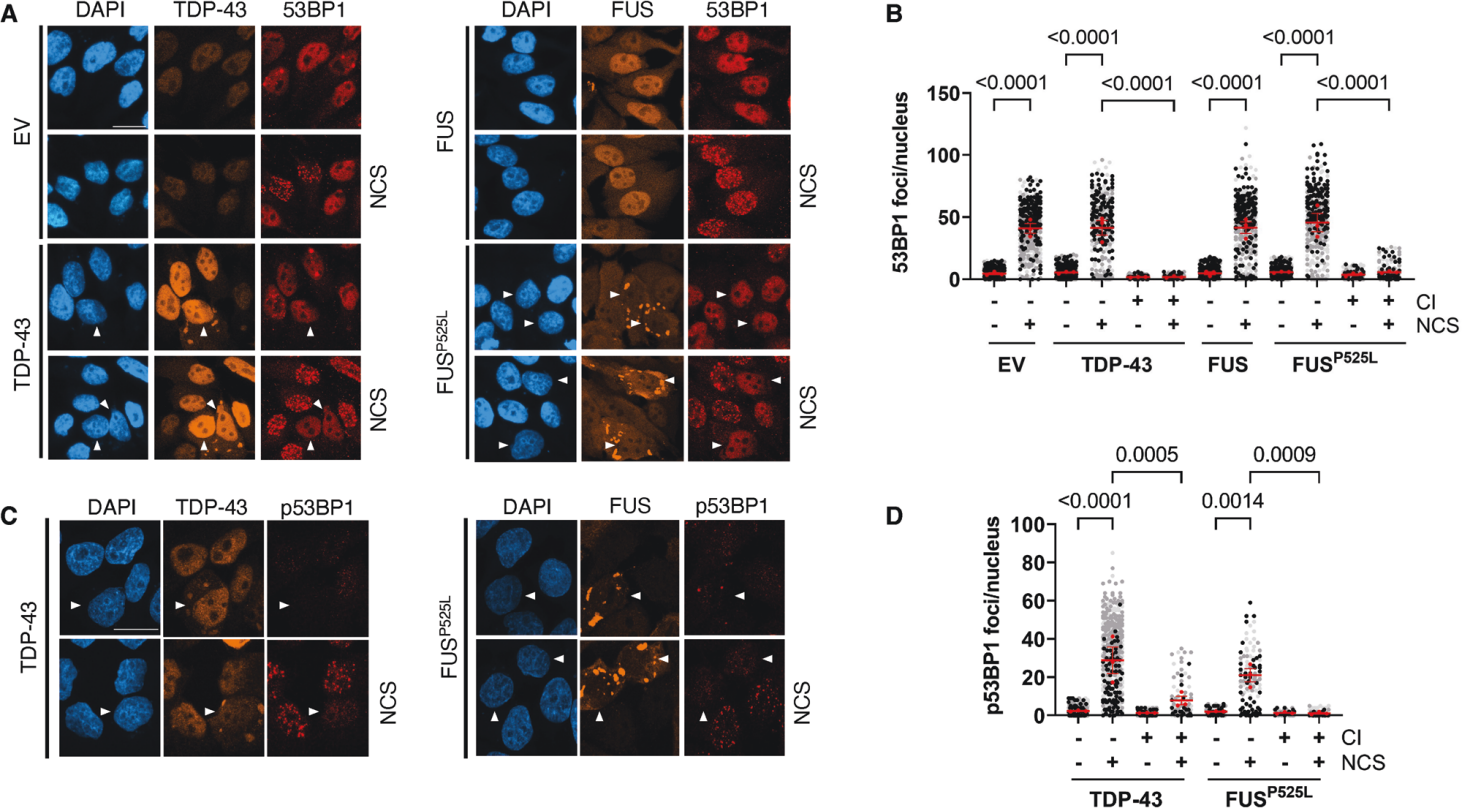
DDR signaling and 53BP1 activation is impaired in cells with TDP-43 and FUS^P525L^ CIs. **A**, **C** Representative images of 53BP1 and p53BP1 (red) in damaged (NCS) or undamaged HeLa cells transfected with plasmids encoding for TDP-43, FUS or FUS^P525L^. Cells transfected with an empty vector (EV) were used as a control. TDP-43 and FUS are shown in orange; nuclei were counter-stained with DAPI. Arrowheads mark cells with CIs; scale bar = 20 µm. **B**, **D** Quantification of DDR marker signals in cells with or without cytoplasmic inclusions (± CI) determined in (**A**, **C**), respectively. The red dots of the super-plots shown in (**B**, **D**) represent the mean values of each biological replicate; red bars indicate the averages ± SEM of three independent experiments.

53BP1 is one of the main mediators of the DDR cascade and it is involved in DNA repair by NHEJ, which is the pathway used by non-replicating cells, including neurons, to repair DSBs [42]. Since we previously observed that DDR initiation, as detected by probing for the auto-phosphorylation of ATM, was altered in cells with both FUS^P525L^ and TDP-43 CIs (Fig. 2C–E), we tested if the recruitment of 53BP1 was also defective in such cells. While NCS-treated cells without CIs mounted 53BP1 foci, cells with FUS^P525L^ or TDP-43 CIs were unable to form 53BP1 foci upon NCS treatment (Fig. 3A, B).

Following DSB formation, 53BP1 is phosphorylated by ATM at multiple S/T-Q motifs to modulate DDR and repair [43, 44]. We therefore monitored by IF 53BP1 phosphorylation in NCS-treated FUS^P525L^- or TDP-43-expressing cells. We observed that CI-containing cells showed significantly less p53BP1 foci compared to those without aggregates (Fig. 3C, D). Differently from what we observed for pATM (Fig. 2C, D), undamaged CI-bearing cells showed low or no p53BP1 signal (Fig. 3C, D). Also, TDP-43 and FUS^P525L^ aggregation did not substantially affect 53BP1 total levels (Fig. S3A), indicating that CIs negatively impacted on 53BP1 activation, rather than on its expression. Overall, these results suggest that the hyper-activation of ATM caused by CI formation is unable to transduce the signal to the downstream DDR mediator 53BP1.

The scaffold protein MDC1 plays a key role in the DSB signaling, ensuring the recruitment of the E3-ubiquitin ligases RNF8 and RNF168, which in turn allows the assembly of 53BP1 at DSBs [45]. Specifically, MDC1 associates to the site of damage mostly in a γH2AX-dependent manner [46] and, differently from 53BP1, functions upstream to both homologous recombination (HR), a repair pathway exploited by replicating cells, and NHEJ [47]. To probe for the impact of CI formation on the ability of MDC1 to localize at DSBs, we stained TDP-43- and FUS^P525L^-expressing cells and their controls for MDC1. Differently from 53BP1, NCS-treated cells with TDP-43 or FUS^P525L^ CIs showed normal damage-induced MDC1 foci formation (Fig. S3B, C).

Taken together, these results indicate that the two DDR mediator proteins, 53BP1 and MDC1, behave differently in cells bearing mutant FUS or TDP-43 CIs, with the profound defect in 53BP1 foci formation not being accompanied by reduced MDC1 focal recruitment.

Since the expression of TDP-43 and FUS^P525L^ triggered the assembly of SGs (Fig. S1G, H), we tested if their formation was sufficient to alter DDR and cause DNA damage accumulation. To this end, we acutely treated HeLa cells with sodium arsenite (NaAsO_2_), an inorganic compound commonly used to promote SG assembly [48]. After incubating the samples with 500 μM NaAsO_2_ for 30 min, nearly all cells exhibited cytoplasmic SGs, as detected by IF against the SG marker TIA-1 (Fig. S3D). Notably, when we examined DNA damage accumulation and DDR activation in these samples, we observed that undamaged (–NCS) NaAsO_2_-treated cells did not show pan-nuclear γH2AX signals (Fig. S3D, E). Moreover, damaged (i.e., with NCS) and NaAsO_2_-treated cells were able to mount a proper response to DSB formation, with 53BP1 foci levels comparable to those of untreated (i.e., without NaAsO_2_) cells (Fig. S3D, E). In summary, these results suggest that SG assembly alone is not sufficient to compromise genome integrity or DDR activation, unlike the formation of cytoplasmic inclusions of TDP-43 or FUS^P525L^.

### Transcription is repressed in cells with TDP-43 or FUS^P525L^ CIs

**Fig. 4:**
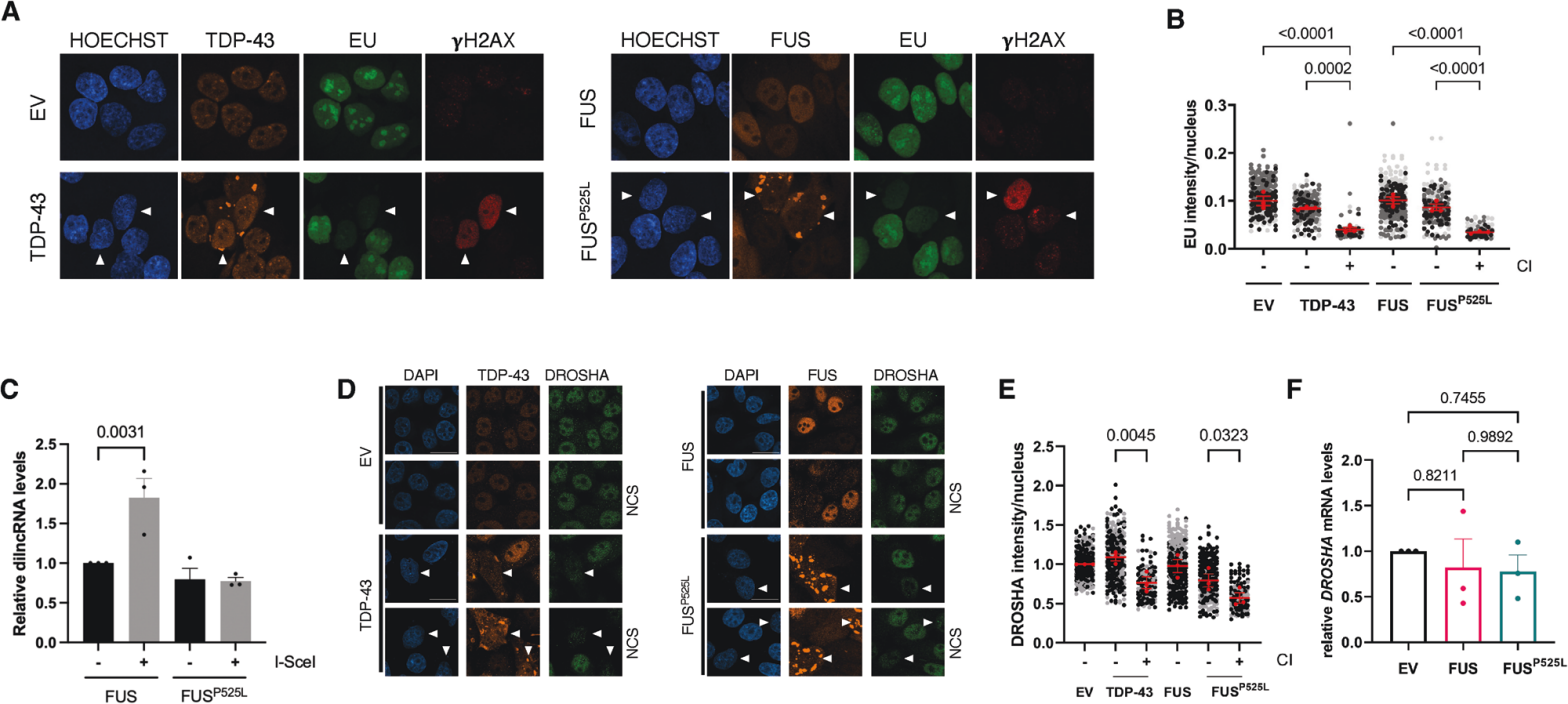
Transcription is halted in cells with TDP-43 and FUS^P525L^ CIs. **A** HeLa cells expressing TDP-43, FUS or FUS^P525L^ were pulse-labeled with EU and analyzed by immunofluorescence; TDP-43 and FUS, EU and γH2AX are shown in orange, green and red, respectively. Nuclear DNA was visualized using Hoechst dye; arrowheads mark cells with CIs. **B** Quantification of EU signal in cells with or without cytoplasmic inclusions (± CI) shown in (**A**). The red dots of the super-plot represent the mean values of each biological replicate; red bars indicate the averages ± SEM of three independent experiments. **C** Damage-induced long non-coding RNA (dilncRNA) expression was studied by strand-specific RT-qPCR in cut (I-SceI +) or uncut (I-SceI -) I-HeLa111 cells selected for the presence of FUS or FUS^P525L^ CIs through FACS. The histogram shows dilncRNA levels normalized over RPLP0 mRNA and shown as relative to the uncut FUS-expressing sample; values are the means ± SEM of three independent experiments. **D** Representative images of DROSHA expression (green) in damaged (NCS) or undamaged HeLa cells transfected with plasmids encoding for TDP-43, FUS or FUS^P525L^; cells transfected with an empty vector (EV) were used as a control. TDP-43 and FUS are labeled in orange; nuclei were counter-stained with DAPI. Arrowheads mark cells with CIs; scale bar = 10 µm. **E** Quantification of DROSHA protein levels in cells with or without cytoplasmic inclusions (± CI) from panel (**D**). The red dots of the super-plot represent the mean values of each biological replicate; red bars indicate the averages ± SEM of three independent experiments. **F** *DROSHA* mRNA levels were monitored by RT-qPCR in HeLa cells selected for the presence of FUS or FUS^P525L^ CIs through FACS. Values are the averages ± SEM of three independent experiments.

TDP-43 and FUS functions have been linked to transcription modulation [28]. Moreover, ongoing transcription is halted in response to DSBs, to allow proper DNA damage resolution and preserve genome integrity [49–52]. Specifically, ATM has been shown to initiate transcriptional repression in response to DSB formation [49]. We thus wondered if the increased levels of DNA damage observed in cells with TDP-43 and FUS CIs also correlated with transcriptional inhibition. To monitor de novo transcription in cells with CIs, we labeled nascent RNAs in TDP-43- and FUS^P525L^-expressing cell cultures with 5-Ethynyl-Uridine (EU) using a “click-chemistry” approach followed by fluorescent microscopy analysis. We observed a significantly lower EU incorporation signal in cells with either TDP-43 or FUS^P525L^ CIs, indicating a global transcriptional repression (Fig. 4A, B).

Since our results indicate that ATM inactivation was sufficient to inhibit γH2AX accumulation in CI-containing cells (Fig. 2A, B), we tested its potential impact on global transcription in cells with CIs. We therefore pharmacologically inhibited the ATM kinase activity in HeLa cells expressing TDP-43 or FUS^P525L^ and monitored nascent RNA synthesis using the assay described above. We observed that ATM inhibition elevated the EU signal in cells with CIs, compared to those treated with the vehicle only (Fig. S4A, B). These results therefore indicate that the accumulation of CIs causes an ATM-dependent repression of global transcription.

### Biogenesis of damage-induced non-coding RNAs is defective in CI-containing cells

Following DSB formation, while pre-existing transcription is repressed, RNAPII is recruited at sites of DNA damage where it drives the local synthesis of damage-induced long non-coding RNAs (dilncRNAs) [35, 53]. Such transcripts can be further processed by DROSHA and DICER to generate DNA damage response RNAs (DDRNAs) that, along with dilncRNAs, control DDR activation and DNA repair [35, 53–55]. We thus investigated if the transcriptional repression observed in CI-containing cells (Fig. 4A, B) also impacted on dilncRNA biogenesis. To accomplish this, we profiled the expression of dilncRNAs in I-HeLa111 cells, where a traceable DSB can be generated at a genomic I-SceI recognition site by expressing the I-SceI meganuclease [56]. To enrich and selectively study dilncRNA levels in cells bearing CIs, we took advantage of the Bimolecular Fluorescence Complementation (BiFC) approach [57]. Specifically, we transfected I-HeLa111 cells with two constructs expressing FUS^P525L^ fused to two distinct fragments of the GFP fluorescent protein, mVenus and mCerulean, that emit a fluorescent signal only when brought into proximity with each other upon FUS protein dimer formation, and even more when FUS aggregates [57] (Fig. S4C). Highly fluorescent cells were in this way enriched by fluorescence-activated cell sorting (FACS) and total RNA was isolated from sorted cells and analyzed for dilncRNA expression by strand-specific RT-qPCR. Upon DSB induction with I-SceI, I-HeLa111 cells expressing WT FUS showed elevated dilncRNA levels, while those expressing FUS^P525L^ failed to induce dilncRNAs following DSB generation (Fig. 4C).

As mentioned above, along with DICER, the endoribonuclease DROSHA participates in DDRNA biogenesis, and associates with DSBs where it is required for 53BP1 recruitment and repair by NHEJ [54, 58–60]. We noticed that cells containing TDP-43 and FUS^P525L^ CIs displayed reduced DROSHA protein levels, compared to those devoid of aggregates (Fig. 4D, E). To determine whether CI formation impacted on *DROSHA* expression at the RNA level, we measured the amounts of *DROSHA* mRNA by RT-qPCR in cells with FUS aggregates using the BiFC system. We observed no change in its levels in samples expressing FUS^P525L^ compared to control cells (Fig. 4F), suggesting that FUS aggregation rather affected DROSHA protein stability. Contrarily, DICER protein levels were not significantly altered in cells with inclusions (Fig. S4D, E).

Taken together, these results indicate that the formation of cytoplasmic aggregates is accompanied by a defective biogenesis of damage-induced non-coding RNAs, important modulators of DDR activation.

### TDP-43 and FUS cytoplasmic aggregation correlates with defective DDR, DNA damage accumulation and reduced DROSHA levels in neuronal lines

**Fig. 5:**
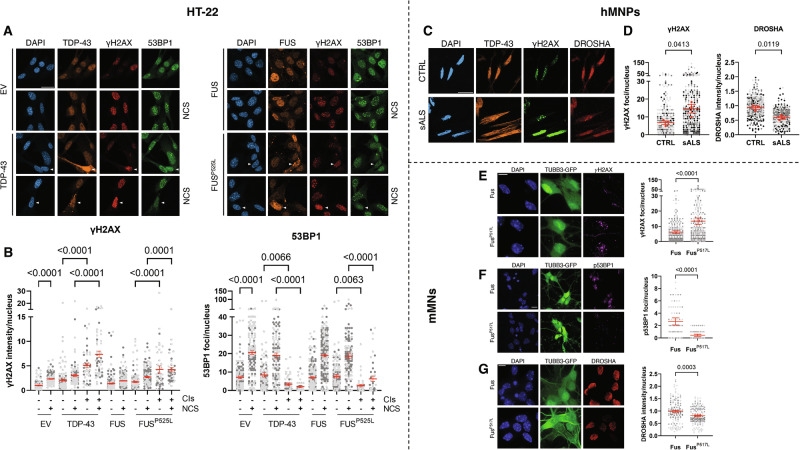
TDP-43 and FUS cytoplasmic aggregation correlates with an altered DDR, DNA damage accumulation and reduced DROSHA levels in neuronal cell lines. **A** Analysis by immunofluorescence (IF) of γH2AX (red) and 53BP1 (green) signals in damaged (NCS) or undamaged HT-22 cells expressing TDP-43, FUS or FUS^P525L^; cells transfected with the empty vector (EV) were also examined. TDP-43 and FUS are shown in orange; nuclei were counter-stained with DAPI (blue); arrowheads mark cells with CIs. Scale bar = 20 µm. **B** Dot-plot showing γH2AX intensity (left) and 53BP1 foci (right) in HT-22 cells with or without cytoplasmic inclusions (± CIs), as determined in (**A**); red bars indicate the averages ± SEM; at least 40 cells were scored for each condition in two independent experiments. **C** Analysis by IF of γH2AX (green) and DROSHA (red) signals in hMNPs derived from sALS or a healthy (CTRL) donor; endogenous TDP-43 (orange) is also shown. Nuclei are counter-stained with DAPI (blue); scale bar = 20 µm. **D** Quantification of γH2AX foci (left) and DROSHA levels (right) in hMNPs, as determined in (**C**). The red dots of the super-plot represent the mean values of each biological replicate; red bars indicate the averages ± SEM of three independent experiments. **E** TUBB3-GFP-expressing mMNs carrying wild-type or mutant Fus were stained for γH2AX. The dot-plot shows γH2AX foci in each nucleus; red bars indicate the averages ± SEM; at least 150 cells were scored for each condition in three independent experiments. **F** IF analysis of p53BP1 foci in mMNs. The dot-plot shows p53BP1 foci in each nucleus; red bars indicate the averages ± SEM. At least 60 cells were scored for each condition in two independent experiments. **G** IF analysis of DROSHA protein levels in mMNs. The dot-plot shows DROSHA intensity in each nucleus; red bars indicate the averages ± SEM. At least 130 cells were scored for each condition in three independent experiments. **E**–**G** nuclei were counter-stained with DAPI; scale bar = 100 µm.

The findings we have presented so far were generated using the HeLa cervical carcinoma cell line. However, TDP-43 and FUS dysfunction are particularly toxic to neurons. Therefore, we examined if the formation of TDP-43 and FUS CIs impacted DDR activation and DNA damage accumulation in neuronal lines as well. To this end, we first employed SH-SY5Y human neuroblastoma cells, which are routinely used as a cellular model to study the molecular mechanisms underlying the development of neurodegenerative diseases [27, 61, 62]. We thus transfected SH-SY5Y cells with plasmids expressing wild-type TDP-43, FUS, or FUS^P525L^, or with an empty vector as a control. The cells were then treated with or without NCS, and DDR activation was monitored in cells containing or lacking CIs. Unfortunately, FUS^P525L^-transfected SH-SY5Y cells displayed very few inclusions (not shown), which prevented us from generating informative and robust results. On the bright side, and as observed in HeLa cells, transfection with the plasmid expressing TDP-43 led to the formation of CIs co-localizing with SGs in SH-SY5Y cells, albeit at a much lower frequency compared to HeLa cells (Fig. S5A, D). Similarly, SH-SY5Y cells containing TDP-43 CIs displayed increased γH2AX levels (Fig. S5B, C). In addition, SH-SY5Y cells bearing TDP-43 CIs and treated with NCS showed reduced 53BP1 foci compared to cells without CIs (Fig. S5B, C). These results confirm that the formation of TDP-43 CIs impairs 53BP1 recruitment and causes DNA damage accumulation also in this neuronal tumor cell line. Additionally, as already shown in HeLa cells (Fig. S2F), TDP-43 CI-containing SH-SY5Y cells displayed reduced Cyclin A expression, suggesting that TDP-43 CI formation impacted on cell cycle progression in these cells as well (Fig. S5D, E). Next, we extended these studies to HT-22 mouse hippocampal neuronal cells. Consistent with the results obtained in HeLa and in SH-SY5Y cell lines, HT-22 cells harboring TDP-43 and FUS^P525L^ CIs exhibited increased γH2AX signals, compared to those lacking CIs (Fig. 5A, B). Moreover, in CI-containing HT-22 cells, 53BP1 focus formation following NCS administration was severely impaired (Fig. 5A, B). In this non-tumoral neuronal line, we also tested if CI formation triggered p53 activation by probing for its phosphorylated form (p-p53^S15^) through IF. We noticed that CI-containing HT-22 cells did not exhibit augmented p-p53^S15^ signals, compared to cells without CIs (Fig. S5F, G). This indicates that CI formation did not cause p53 activation.

Then, we sought to validate our results in other relevant neuronal lines, namely human motor neuron progenitors (hMNPs) and mature murine motor neurons (mMNs). hMNPs were generated from induced pluripotent stem cells (iPSCs) of a sALS patient or a healthy donor (Fig. S5H) [63]. sALS hMNPs, characterized by a marked TDP-43 cytoplasmic delocalization, showed augmented γH2AX signals and reduced DROSHA levels, compared to control hMNPs (Fig. 5C, D). mMNs carrying on both alleles the FUS^P517L^ mutation, which is equivalent to human FUS^P525L^, present a strong FUS signal in the cytoplasm, whereas FUS is almost exclusively localized to the nucleus of wild-type mMNs (Fig. S5I) [64]. Notably, FUS^P517L^-expressing mMNs exhibited increased γH2AX, reduced p53BP1 foci, and diminished DROSHA signals with respect to control mMNs (Fig. 5E–G).

In summary, these results confirm that alterations of TDP-43 and FUS subcellular distribution are associated to dysfunctional DDR, accumulation of DNA damage and decreased DROSHA levels in neuronal cells as well.

### Enoxacin restores proficient DDR, reduces DNA damage accumulation, and partially restores transcription in CI-bearing cells

**Fig. 6:**
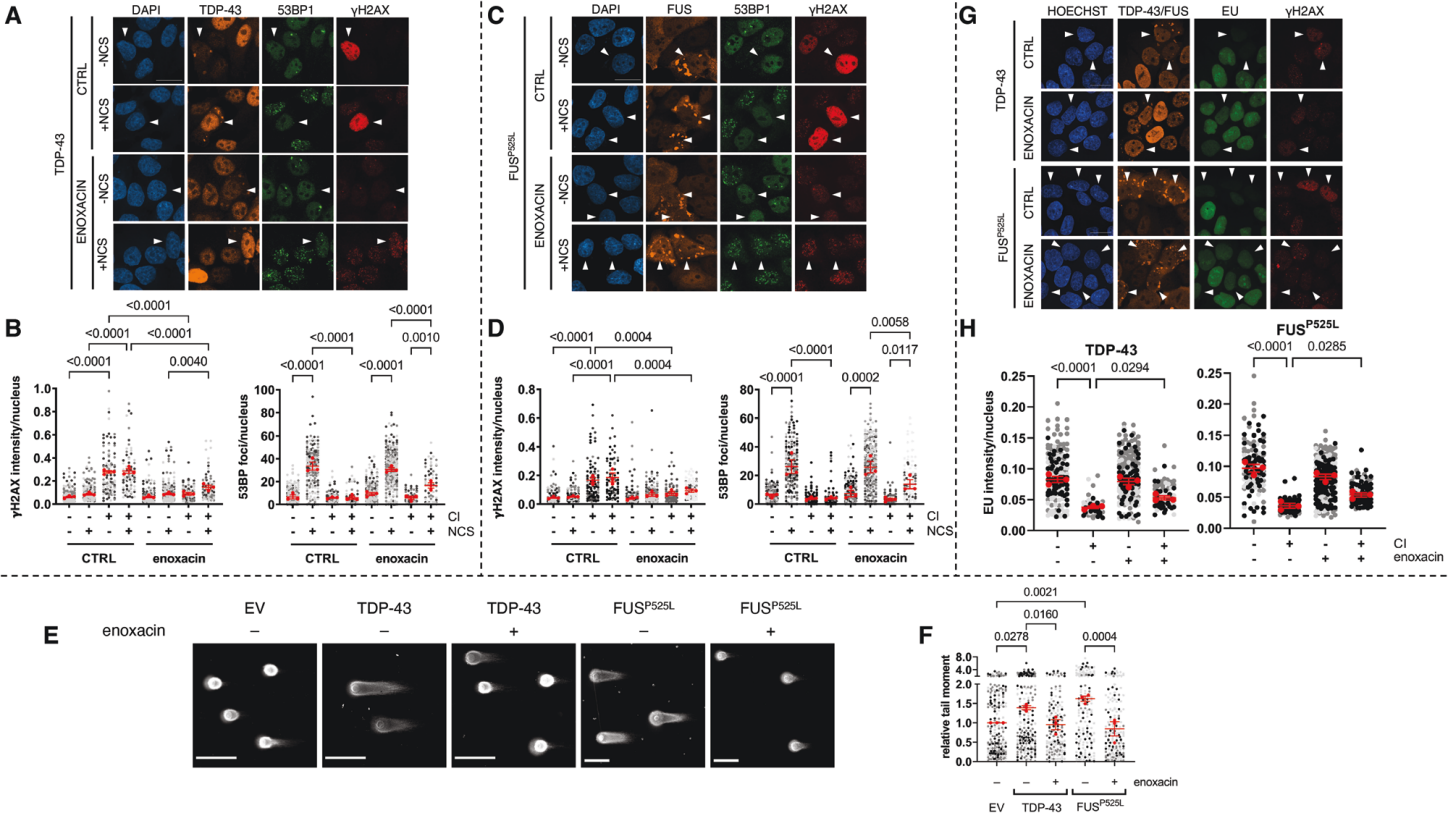
Enoxacin rescues foci formation and partially restores transcription in cells with TDP-43 and FUS^P525L^ CIs. **A**, **C** HeLa cells expressing TDP-43 or FUS^P525L^ were treated or not with enoxacin prior to NCS administration and analyzed by immunofluorescence for DDR activation; TDP-43 and FUS are labeled in orange. Nuclei were counter-stained with DAPI; arrowheads mark cells with CIs. Scale bar = 10 µm. **B**, **D** Quantification of γH2AX intensity and 53BP1 foci in cells with or without cytoplasmic inclusions (±CI) shown in (**A**, **C**), respectively. The red dots of the super-plots represent the mean values of each biological replicate; red bars indicate the averages ± SEM of three independent experiments. **E** Representative images of comet assay experiments performed in undamaged HeLa cells transfected with the plasmid expressing TDP-43, FUS^P525L^, or an empty vector (EV) and treated or not with enoxacin; scale bar = 100 µm. **F** Tail moment analysis of HeLa cells shown in E. The red dots of the super-plot represent the mean values of each biological replicate; red bars indicate the averages ± SEM of three independent experiments. **G** HeLa cells expressing TDP-43 or FUS^P525L^ were treated or not with enoxacin and pulse-labeled with EU prior to immunofluorescence analysis; TDP-43 and FUS are shown in orange. Nuclear DNA was visualized using Hoechst dye; arrowheads mark cells with CIs. Scale bar = 10 µm. **H** Quantification of EU signal in cells with or without cytoplasmic inclusions (±CI) determined in (**G**). The red dots of the super-plot represent the mean values of each biological replicate; red bars indicate the averages ± SEM of three independent experiments.

Enoxacin is a small molecule able to enhance the enzymatic activity of the endoribonuclease DICER, involved in the processing of different classes of short RNAs, including small interfering RNAs (siRNAs) and miRNAs [65, 66]. In this regard, enoxacin treatment has been shown to promote the biogenesis of DICER-dependent small RNAs [65, 66] and we previously demonstrated it stimulates DDR signaling and DNA repair in cultured cells [55, 61, 67, 68]. Therefore, we tested if enoxacin administration could restore proper DDR activation and reduce DNA damage accumulation in cells with CIs. HeLa cells were treated with enoxacin for 48 h prior to transfection with TDP-43 or FUS^P525L^ expressing plasmids and monitored for 53BP1 and γH2AX signals by IF. Enoxacin-treated cells bearing TDP-43 and FUS^P525L^ CIs showed both increased 53BP1 foci and reduced γH2AX levels, compared to untreated samples (Fig. 6A–D). In addition, supplementing enoxacin to cells expressing TDP-43 or FUS^P525L^ decreased DSBs back to normal levels, as revealed by comet assay (Fig. 6E, F). These results therefore demonstrate that enoxacin can restore DDR function and reduce DNA damage accumulation in cells with CIs.

Since transcription was inhibited in CI-containing cells (Fig. 4A, B), we tested whether enoxacin administration could also restore proficient transcription in these cells. Thus, we performed the EU incorporation assay described above in enoxacin-treated cells expressing either TDP-43 or FUS^P525L^ and we observed that enoxacin treatment significantly increased the levels of EU incorporation in cells harboring CIs, in comparison to untreated CI-bearing cells, although not to the level detected in cells devoid of CIs (Fig. 6G, H).

Altogether, these results show that enoxacin improves DDR and DNA repair in cells with TDP-43 or FUS^P525L^ aggregates, leading to restored global transcription.

### Enoxacin stimulates DDR and lowers DNA damage accumulation in an ALS murine model of FUS pathology

**Fig. 7:**
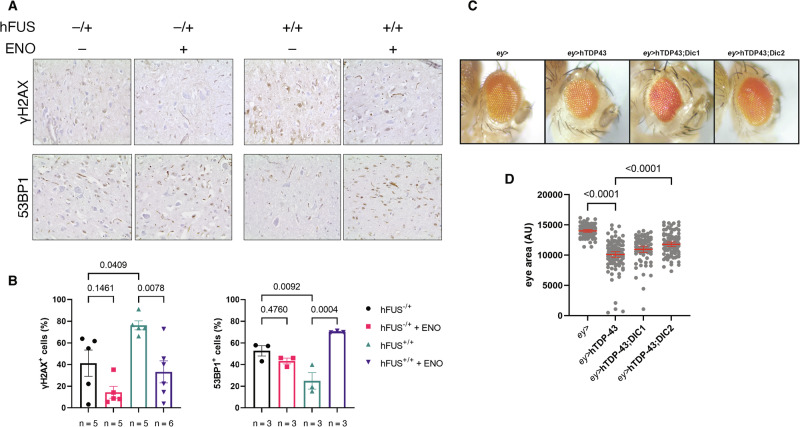
Enoxacin stimulates DDR and reduces DNA damage accumulation in hFUS murine spinal cords. Dicer-2 overexpression counteracts retina degeneration in hTDP-43 *Drosophila melanogaster* eyes. **A** Representative IHC images of spinal cords of hFUS-expressing mice, treated with enoxacin or vehicle and stained for γH2AX and 53BP1. **B** Quantification of the percentage of cells positive for the indicated markers as determined in (**A**). Values are the means ± SEM. At least three mice were studied for each condition; the precise number is indicated below each histogram. **C** Representative images of *Drosophila melanogaster* eyes from adult flies expressing the indicated constructs under control of eyeless-GAL4. **D** Dot plot showing the quantification of mean eye area of the flies with the indicated genotypes; more than 70 eyes per genotype were analyzed in three independent experiments; red bars represent the means ± SEM.

We then sought to extend and validate the observations obtained in cultured cells in in vivo models of ALS pathology. To this aim, we administrated enoxacin to either non-pathological heterozygous or pathological homozygous mice over-expressing the wild-type human homolog of FUS (hFUS) [69, 70] and probed for DDR activation and damage accumulation, by staining murine spinal cords for γH2AX and 53BP1 by immunohistochemistry (IHC) technique. Consistent with our results in cultured cells, hFUS homozygous mice presented higher levels of γH2AX and reduced 53BP1 signals in their spinal cord, with respect to heterozygous animals (Fig. 7A, B). Importantly, in enoxacin-treated homozygous mice we observed that the signal of 53BP1 increased, whereas γH2AX levels decreased, compared to vehicle-treated animals (Fig. 7A, B).

Taken together, these exciting results suggest that enoxacin administration in vivo in mice with FUS pathology was effective in stimulating DDR, reducing DNA damage accumulation.

### DICER overexpression counteracts neurodegeneration in a *Drosophila melanogaster* ALS model system

Our results in mice show that the enhancement of DNA repair through enoxacin administration reduces DNA damage accumulation in vivo. However, how this can impact on neurodegeneration remains unexplored. To test this, we used a *Drosophila melanogaster* model of TDP-43-mediated neurotoxicity, in which the ectopic expression of the human TDP-43 (hTDP-43) in the fly ommatidia causes progressive retinal degeneration, as shown by a significant reduction of the eye area, which is not associated to a diminished proliferation of ommatidial cells in the larvae [71] (Figs. 7C, D, S6). To mimic the effects of enoxacin administration in this system, we overexpressed both *D. melanogaster* isoforms of Dicer in hTDP-43-expressing flies and monitored their impact on retinal degeneration. Differently from mammals, the *D. melanogaster* genome encodes for two distinct Dicer enzymes: Dicer-1, mainly involved in cytoplasmic miRNA biogenesis, and Dicer-2 with a more prominent role in siRNA processing in the nucleus [72]. Remarkably, the overexpression of Dicer-2 significantly rescued the eye area in hTDP-43-expressing flies (Fig. 7C, D). Conversely, no major effects were observed in flies co-expressing hTDP-43 and Dicer-1, ruling out a central role for miRNAs in preventing retinal degeneration (Fig. 7C, D).

These results indicate that stimulating Dicer functions may be beneficial in counteracting TDP-43-mediated neurodegeneration.

## Discussion

Accumulation of unrepaired DNA damage and the resulting disruption of genome integrity can have profound consequences, including ageing and inflammation, which in turn increase the susceptibility to various pathological conditions, especially neurodegenerative diseases [25, 73, 74]. In light of this, mounting evidence have recently put into relation DNA damage repair defects with neurodegeneration in ALS [25]. ALS is characterized by an aberrant accumulation of insoluble cytoplasmic inclusions (CIs) containing TDP-43 and other RNA-binding proteins, including FUS [2]. Interestingly, endogenous wild-type FUS and TDP-43 have been reported to play crucial roles in DNA damage repair [75]: FUS associates with DNA lesions by interacting with PAR-chains generated at damaged chromatin [76], and favors DNA damage resolution by recruiting the XRCC1/LIG3 repair complex to DNA lesions [77]. In addition, recently, TDP-43 has been implicated in DNA repair and reported to associate at DSBs with factors of the NHEJ machinery, ultimately contributing to DNA damage resolution in physiological conditions [62]. These studies have highlighted a causative link between the depletion of FUS and TDP-43 with genome instability and reduced survival in neuronal cells [62, 77]. Nonetheless, the impact of TDP-43 and FUS cytoplasmic aggregation—a pathological hallmark of ALS—on DNA damage accumulation has not been fully elucidated.

Here, we show that the deposition of TDP-43 and FUS^P525L^ CIs, which are associated with stress granules (SGs) (Fig. S1G, H), triggers a dysfunctional DDR (Figs. 2C–E, 3 and 5A, B), and induces DSB accumulation (Fig. 1). Differently, cells overexpressing FUS or TDP-43, but devoid of CIs, do not accumulate DNA damage (Figs. 1A, B and 5A, B) and display proficient DDR when exogenous DNA damage is inflicted (Figs. 2C–E, 3 and 5A, B). Specifically, cells accumulating CIs display a hyper-activation of the apical DDR kinase ATM, responsible for the massive increase of the pan-nuclear γH2AX signal observed (Fig. 2). Such aberrant activation of ATM is however unable to transduce the signal to the downstream DDR and repair factors, including 53BP1 (Fig. 3), ultimately hampering their recruitment to DNA damage sites and consequently preventing DNA damage resolution through NHEJ, the predominant DNA repair pathway in non-dividing cells. Importantly, ATM inhibition using small molecules prevents the accumulation of DNA damage signals in cells with CIs (Fig. 2A, B). Interestingly, ATR inactivation had a little but significant impact in reducing γH2AX levels in cells with TDP-43 CIs, but not in those containing FUS^P525L^ inclusions (Fig. 2A, B). Such discrepancy can be explained by the fact that dysfunctional TDP-43 has been associated with DNA replication defects, possibly leading to ATR activation [38]. Consequently, ATR may contribute, at least partly, to the augmented γH2AX signal observed in cells containing TDP-43 CIs. We also noticed that the induction of SG formation by acute treatment with NaAsO_2_ was not sufficient to cause a DDR defect or DNA damage accumulation (Fig. S3D, E). This observation is consistent with the finding that acute administration of NaAsO_2_ to ALS fibroblasts leads to the formation of SGs lacking TDP-43, whereas a prolonged NaAsO_2_ exposure induces the assembly of SGs containing TDP-43 inclusions [78]. Our results therefore indicate a causative relationship between TDP-43 and FUS inclusions in SGs and genotoxicity.

Shutdown of global transcription is one of the immediate consequences of DNA damage generation [79]. In particular, the activation by auto-phosphorylation of ATM in response to DNA damage has been shown to enforce chromatin modifications surrounding DSBs, which in turn prevent RNAPII elongation [49]. Loss of either TDP-43 and FUS has been linked to transcription arrest and consequent formation of DNA damage at sites of active transcription [28]. Consistent with this, cells harboring TDP-43 or FUS^P525L^ CIs display dysfunctional ATM hyperactivation (Fig. 2C–E) and pan-nuclear γH2AX signals (e.g., Figs. 1A, B, 2A, B and 4A) and exhibit reduced global transcription (Fig. 4A, B). Pharmacological inactivation of ATM rescues proficient RNA synthesis in CI-containing cells (Fig. S4A, B). Overall, our results indicate that TDP-43 or FUS^P525L^ CI formation, by inducing aberrant DDR and DSB generation, hampers transcription. In this regard, we noticed that CI formation does not engage p53 activation, as determined by probing for its ATM/ATR-dependent phosphorylation at serine 15 (p-p53) through immunofluorescence in HT-22 cells (Fig. S5F, G). Moreover, TDP-43 and FUS aggregation caused cell cycle arrest and transcriptional inhibition also in HeLa cells, which express very low levels of p53 [80]. Our data therefore suggest that the events observed following CI formation occur in a p53-independent manner. Notably, CI-induced transcriptional repression may have profound implications particularly in neurons, where longer transcripts are enriched with respect to other cell types [81, 82]. In this regard, the inactivation of genes involved in regulating RNAPII processivity, by favoring the accumulation of truncated transcripts, has been associated to synaptic dysfunction, autism, and neurodegeneration [82]. Such findings, along with the results shown here, provide an intriguing explanation for the higher sensitivity of neural tissues to CI accumulation, compared to others.

Interestingly, the formation of cytosolic aggregates not only hampers the synthesis of damage-induced non-coding RNAs (Fig. 4C), but it might also impair their processing into DNA damage response RNAs (DDRNAs) by reducing the nuclear levels of DROSHA (Fig. 4D, E). Such events may ultimately interfere with faithful DNA damage resolution [35, 53, 60, 83], likely establishing a detrimental feedback loop that further exacerbates the accumulation of unrepaired DNA lesions. Accordingly, in this double-hit process, even minimal improvements in DDRNA biogenesis may exert beneficial effects.

The small molecule enoxacin, which was reported to enhance DICER activity [66], is one of the few compounds able to boost DDR and improve repair [55, 61, 67, 68]. Its administration was demonstrated effective in improving DNA damage resolution in irradiated primary mouse cortical neurons [55] and in an AD model of human neuronal cells [61]. In the present study, we show that stimulating DICER activity through enoxacin restored functional DDR and decreased DNA damage accumulation in HeLa cells with TDP-43 and FUS^P525L^ CIs, and in vivo in hFUS-expressing mice (Figs. 6A–F and 7A, B). To evaluate whether the observed effects of DICER enhancement on stimulating DDR and repair were also beneficial at counteracting neurotoxicity, we expressed both Dicer-1 and Dicer-2 in a *Drosophila melanogaster* model of TDP-43-mediated retinal neurodegeneration. Intriguingly, while the overexpression of Dicer-2 almost completely restored the normal fly eye size, Dicer-1 ectopic expression had no impact (Fig. 7C, D). This suggests that the small RNAs generated by DICER exert their function in controlling DDR and repair, ultimately promoting neuronal cell survival through an siRNA-mediated mechanism, rather than in a miRNA-like fashion.

In conclusion, our results strongly suggest that the factors involved in DDR may represent relevant pharmacological targets for the treatment of the cytotoxic effects caused by TDP-43 and FUS CIs.

## Materials and methods

### Cell culture and treatments

Cell lines were authenticated by STR profiling (GenePrint system, Promega) and tested for mycoplasma contamination. HeLa, FUCCI-HeLa and HT-22 cells were cultured in DMEM supplemented with 10% FBS, 1% L-glutamine and 1% penicillin/streptomycin (P/S). SH-SY5Y were cultured in DMEM/F12 supplemented with 15% FBS and 1% P/S. DNA damage was induced by treating cells with 50 ng/mL neocarzinostatin (NCS) (Sigma, #N9162) for 20 min at 37 °C. Stress granule formation was induced by treating HeLa cells with 500 μM of sodium arsenite (NaAsO_2_) for 30 min at 37 °C. I-HeLa111 were cultured in DMEM supplemented with 10% tetracycline-free FBS, 1% L-glutamine and 1% P/S and selected regularly with G418 (1 mg/ml) and hygromycin B (300 μg/ml). In I-HeLa111, I-SceI-mediated double-strand breaks were generated by doxycycline administration (1 μg/ml) for at least 16 h. To inhibit ATM and ATR, cells were treated with 5 µM KU60019 and 10 µM VE-821 overnight, respectively. Where indicated, HeLa cells were treated with 50 µM enoxacin (Millipore, #557305-250MG) for 24 h prior to plasmid transfection and left until the end of the experiment (48 h in total).

### Transfections

In total, 1–2 μg of each plasmid was transfected for 24 h using Lipofectamine 2000 (Invitrogen; #11668-027) according to the manufacturer instructions. The following plasmids were employed: pcDNA3.1+ (ThermoFisher) was used as an empty vector negative control; MYC-tagged WT TDP-43 expressing vector, a kind gift of Dr. Emanuele Buratti on concession of Dr. Leonard Petrucelli (Mayo Clinic, Jacksonville, Florida); FLAG-tagged WT FUS and FUS^P525L^ expressing plasmids. For the Bimolecular Fluorescence Complementation (BiFC) experiments, WT FUS or FUS^P525L^ were cloned in pCS2plus-mCerulean 156-239-GGGS (Addgene, #162616) and pCS2plus-mVenus1-155-GGGS (Addgene, #162610), both gift from Marco Morsch [57], using the Gibson Assembly kit (ThermoFisher). 0.7 µg of each vector were transfected using Lipofectamine 2000.

### Immunofluorescence and image analysis

Immunofluorescence (IF) for DDR markers was performed as already described [54]. IF for TDP-43 and FUS was conducted with antibodies against the endogenous proteins (Supplementary Table 1), unless otherwise specified. Images were acquired using a confocal linear laser-scanning microscope (Zeiss LSM 800). Quantitative immunofluorescence analyses were performed in parallel with identical acquisition parameters and exposure times among conditions, using CellProfiler software [84]. To distinguish between cells with or without CIs, a unique number was assigned to each cell nucleus in every image with CellProfiler. DICER cytoplasmic signal was quantified using Fiji software (NIH). For a complete list of the antibodies used in this study see Supplementary Table 1.

Colocalization of TDP-43 and FUS with TIA-1 or G3BP1 was determined using JACoP plugin (http://rsb.info.nih.gov/ij/plugins/track/jacop.html) of Fiji software [85]. We calculated Manders’ overlap coefficient (MOC), which represents the fraction of TDP-43 or FUS signals overlapping with TIA-1 or G3BP1 signals. The best-fit lower threshold was determined using the threshold tool and visually inspected. We considered the MOC of at least two biological replicates and performed at least 15 acquisitions per sample per replicate.

### Immunoblotting

Immunoblotting analyses were conducted as shown previously [60]. Protein amounts were quantified using Image Lab 6.1 software (Bio-Rad). For a complete list of the antibodies used in this study see Supplementary Table 1. The full length uncropped original western blots used in this work are provided in the ‘Supplemental Material’ file.

### Comet assay

To detect DSB formation in cultured cells, we performed a neutral comet assay as previously described [55].

### BrdU incorporation assay

After plasmid transfection, cells were treated with 10 µM BrdU (MedChemExpress, #HY-15910/CS-2028) overnight and incubated at 37 °C. The following day, cells were washed twice in 1X PBS and fixed in 4% paraformaldehyde for 10 min at RT. Coverslips were then washed twice in 1X PBS and blocked in 1X PBG (0.5% bovine serum albumin, 0.2% gelatin from cold water fish skin (Sigma Aldrich, #G7765) in 1x PBS) for 1 h at RT. Next, coverslips were incubated for one hour at RT in a humid chamber with a 1X PBG mix containing an anti-BrdU primary antibody (BD Laboratories, #347580) diluted 1:20 and RQ1 DNAse (Promega, #M610A) and RQ1 DNAse buffer (Promega, #M198A), each diluted 1:10. After incubation of the primary antibody, cells were washed three times in 1X PBS and immunofluorescence continued as described above.

### EU incorporation

5-EU incorporation experiments were performed as already described [86]. Briefly, following DNA damage generation via NCS treatment, nascent RNAs were labeled with 1 h pulse of 1 mM 5-EU. Cells were then fixed in 4% paraformaldehyde (PFA; ChemCruz; #sc-281692) and levels of EU incorporation were detected by Click-iT RNA imaging kit (Invitrogen; #C10330) according to the manufacturer’s instructions.

### Cell sorting

24 h post plasmid transfection, cells were washed twice with 1X PBS, gently scraped and collected in 1 mL of sorting buffer (2 mM EDTA, 20 mM HEPES pH 7.3 in 1X PBS) per condition, collected in 15 mL tubes and placed on ice. Next, samples were transferred onto a 5 mL falcon equipped with a 35 µM nylon mesh (Corning, #3532235) and vortexed thoroughly to dissociate any cell clusters that might have formed. Next, samples were analysed on an S3e Cell sorter (BioRad). 30000 cells transfected with the EV were analysed first to set the gates. Next, cells expressing WT or FUS^P525L^ were sorted in purity mode and the positive cells were collected. Following cell sorting, cells were centrifuged at 4 °C for 1.5 min at full speed and the supernatant was discarded. Cells were then either lysed to collect RNA or frozen at –80 °C for future use.

### RT-qPCR

Total RNA extraction was performed using the miRNeasy Mini Kit (Qiagen, #1038703), according to the manufacturer’s instructions. 1 µg of total RNA was reverse transcribed using the SuperScript IV First-Strand Synthesis System (Invitrogen, #18091050). For dilncRNA detection in I-HeLa111 cells, 500 ng of total RNA were reverse transcribed with the SuperScript III First-Strand Synthesis System (Invitrogen, #18080-51) using gene-specific primers. Then, samples were chilled on ice and incubated for 20 min at 37 °C with 1 µL of RNase H (Invitrogen). Real-time quantitative PCR (qPCR) reactions were performed on a LightCycler® 480 II Sequence Detection System (Roche) using QuantiTect SYBR® Green PCR kit (Qiagen; #204145). For a complete list of primers used in this study see Supplementary Table 2.

### Human motor neuron progenitors (hMNPs)

Human neural stem cells (hNSCs) obtained from sex- and age-matched control and sporadic ALS iPSCs were differentiated into hMNPs as previously reported [63]. Briefly, hNSCs cultured in adhesion conditions were differentiated into hMNPs in 7 days using a medium composed of 2× Neurobasal (ThermoFisher Scientific, USA), 2× Advanced DMEM/F12, 50× Neural Induction Supplement (ThermoFisher Scientific, USA), 0,1 µM Retinoic Acid (RA) (Sigma-Aldrich, USA) and 0,5 µM Purmorphamine (ThermoFisher Scientific, USA).

### Generation and analysis by immunofluorescence of mouse motor neurons (mMNs)

mMNs carrying wild-type or mutant FUS on both alleles were differentiated from mouse embryonic stem cells as described before [64]. Cells cultured for 2 days after embryoid body dissociation were replated on HCl 1 M, pre-washed and pre-coated glass coverslips (0.01% poly-L-ornithine, 20 mg/mL laminin) and fixed in 4% PFA (Electron Microscopy Sciences) /4% sucrose/5 mM MgCl2/1 X PBS for 30 min at 4 °C. Cells were then washed once in 2% sucrose/1 X PBS and then twice in 4% sucrose/1 X PBS. Following the dehydration steps with the room temperature ethanol (50%, 70%), cells were stored at +4 °C in absolute ethanol + 1:100 VRC (Sigma-Aldrich) until use or processed immediately. Cells were permeabilized 0.2% (0.25% for p53BP1 IF) Triton X-100 for 20 min at room temperature, blocked in 1% goat/1% donkey serum (2% BSA for p53BP1 IF)/PBS for 1 h and then incubated with anti-γH2AX (1:1000, Ab11174, Abcam), anti-phospho-53BP1 (1:500, 3364, Cell signaling), anti-DROSHA (1:500, 3364, Cell Signaling) or rabbit polyclonal anti-TLS/FUS (1:300, ab84078, Abcam) and anti-beta III tubulin (1:200, AB9354, Sigma-Aldrich) primary antibodies overnight at 4 °C. Subsequently, they were incubated with donkey anti-rabbit Alexa Fluor Plus 647 (A32795; Thermo Fisher Scientific) and goat anti-chicken Alexa 488 (SAB4600031, Sigma-Aldrich) secondary antibodies diluted 1:200 in 1% goat/1% donkey serum (2% BSA for p53BP1 IF)/PBS for 45 min at room temperature. After washing twice in 1X PBS, cells were incubated with DAPI solution for 5 min at room temperature and then mounted using ProLong Diamond Antifade Mountant. mMNs samples were imaged using an inverted confocal Olympus IX73 microscope equipped with a Crestoptics X-LIGHT V3 spinning disk system and a Prime BSI Express Scientific CMOS camera and with an Olympus iX83 FluoView1200 laser scanning confocal microscope. The acquisitions were obtained using a UPLANSApo 60X (NA 1.35) oil objective and a UPlanSApo 100× (NA 1.40) oil objective and were collected with the MetaMorph software (Molecular Devices). The z stack confocal microscopy images were taken automatically (200 nm Z-spacing) and merged with the maximum intensity projection method.

### Mice

Mice were housed at the Tor Vergata University Animal Facility (CIMETA) in accordance with the FELASA Recommendations, European Guidelines for the use of animals in research (2010/63/EU), and Italian Laws (D.L. 26/2014). They were kept at constant temperature of 22 ± 1 °C, relative humidity of 50%, and a 12-h light cycle (7 a.m.–7 p.m.), with free access to food and water. To ensure nutrition and hydration, wet food was provided to cages where mice displayed signs of paralysis. Hemizygous Tg (Prnp-FUS) WT3Cshw/J mice expressing human wild-type FUS (hFUS ±, Jackson Laboratories), displaying no pathological signs, were backcrossed to obtain homozygous mice (hFUS+/+), models of ALS. Genotyping was performed as previously described [69]. All experiments were conducted in compliance with the ARRIVE guidelines, with the approval by the local ethics committee and the Italian Ministry of Health. Every effort was made to minimize mice suffering and reduce the number of mice used to obtain reliable results. A total of *n* = 12 hFUS mice (sex-mixed groups) were included in the study. They were treated intraperitoneally daily with either enoxacin at a dose of 10 mg/kg or with vehicle. Enoxacin (Millipore, #557305-250MG) was initially dissolved in 1 M NaOH solution to a concentration of 312 mM and then diluted 100× in PBS for injection. Vehicle was 10 mM NaOH in PBS. Treatment was initiated at about 26 days, which correspond to early symptoms onset in hFUS +/+ mice, for the following 8 days, until mice reached an advanced symptomatic stage (about 34 days). Mice were euthanized with CO_2_, spinal cords were removed and fixed in a 4% paraformaldehyde (PFA) for 12 h and finally immersed in a 30% sucrose solution in PBS.

### Immunohistochemistry

Immunohistochemistry staining was conducted as shown before [87]. For each section studied, five non-overlapping fields were analyzed using the segmentation-based algorithm Multiplex IHC v3.2.3 of HALO software (Indica Labs).

### Fly strains

*Drosophila* stocks and crosses were maintained on *Drosophila* standard medium (Nutri-fly, Genesee Scientific) at 25 °C. UAS-hTDP-43 fly line was gifted from Fabian Feiguin. Bloomington stock center (http://flybase.bio.indiana.edu/) provided the GAL4 driver and the other strains utilized: eyeless-GAL4 inserted on the second chromosome (5534 (w[*]; P{w[+m*]=GAL4-ey.H}3-8); both Dicer transgenes were inserted on the third chromosome (59022 (w*;P{UAS-Dcr-2.D}2,P{UAS-mCherry.CAAX.S}2;TM2/TM6B,Tb1)), and (36510 (y1w*;P{UAS-Dcr-1.D}3).

### Analysis of Drosophila melanogaster eyes

Pictures of fly eyes were taken with the stereomicroscope (ZEISS Semi 508, 50X) equipped with Axiocam camera 105 and exploiting ZEISS ZEN software (blue edition). Eye area was measured with the open-source software NIH ImageJ FIJI.

### Drosophila eye disc labeling

Third instar eye discs were prepared, stained, and mounted following previously described protocols [88]. Briefly, larval brains were isolated by grabbing the mouth hooks with one pair of forceps while gently pulling the rest of the body with another pair of forceps, in a drop of physiological saline buffer. The eye and antennal imaginal discs remained attached to the brain via the hooks, which were used to transfer the samples into the fixation medium. Larval tissues were fixed for 1 h in fixation buffer (4% paraformaldehyde, 4% sucrose in phosphate buffer), permeabilized with permeabilization buffer (0,25% Triton-X in 1× PBS), and blocked in blocking solution (0,1% Triton-X, 3% BSA in 1× PBS). The following antibodies were used: anti-phospho-Histone H3 (Ser10) (Merck), diluted 1:500 was incubated overnight; donkey anti-rabbit Oregon Green (Jackson Immunoresearch), diluted 1:300. Immunofluorescence analysis was performed using an Axiophot epifluorescence microscope (Carl Zeiss), and fluorescence images were processed with Adobe Photoshop.

### Statistical analysis

We did not use any criteria to determine the sample size. As much data as possible was collected depending on the nature of the experiments or in order to have statistical analysis. For animal studies, no statistical methods were used to estimate the sample size. For each experiment, we performed at least three biological replicates, unless stated otherwise; this information is reported in the figure legends.

For in vitro experiments, wells were randomly assigned into each group, and all cells were analyzed equally; for in vivo experiments in mice, animals were randomized to the experimental groups. No blinding method was applied, as we used unbiased software for data collection and analysis.

Prism 9 software (GraphPad) was used to generate graphs, perform statistical analysis and occasionally remove values with the Robust regression and Outlier removal method. Statistical analysis was performed using ordinary One-way ANOVA test, assuming equal SDs between the analyzed groups; unpaired two-tailed *t-*test was applied in Figs. 5D–G and S6B. The exact p-values, center values, and errors are indicated in each figure and its legends.

## Supplementary information

Supplementary Figures 1-6

Supplementary Figure Legends

Supplementary Tables

Original data files

## Source data

Source data

## Data Availability

Source data are provided with this paper and available online. All other data that support the findings of this study are available from the corresponding authors on reasonable request.
