# Supplementary figures and images for: DNA damage response defects induced by the formation of TDP-43 and mutant FUS cytoplasmic inclusions and their pharmacological rescue
Source: Cell Death Differ. 2025 May 29;32(12):2309–22. doi: 10.1038/s41418-025-01530-7 (PMC12669588; doi:10.1038/s41418-025-01530-7)

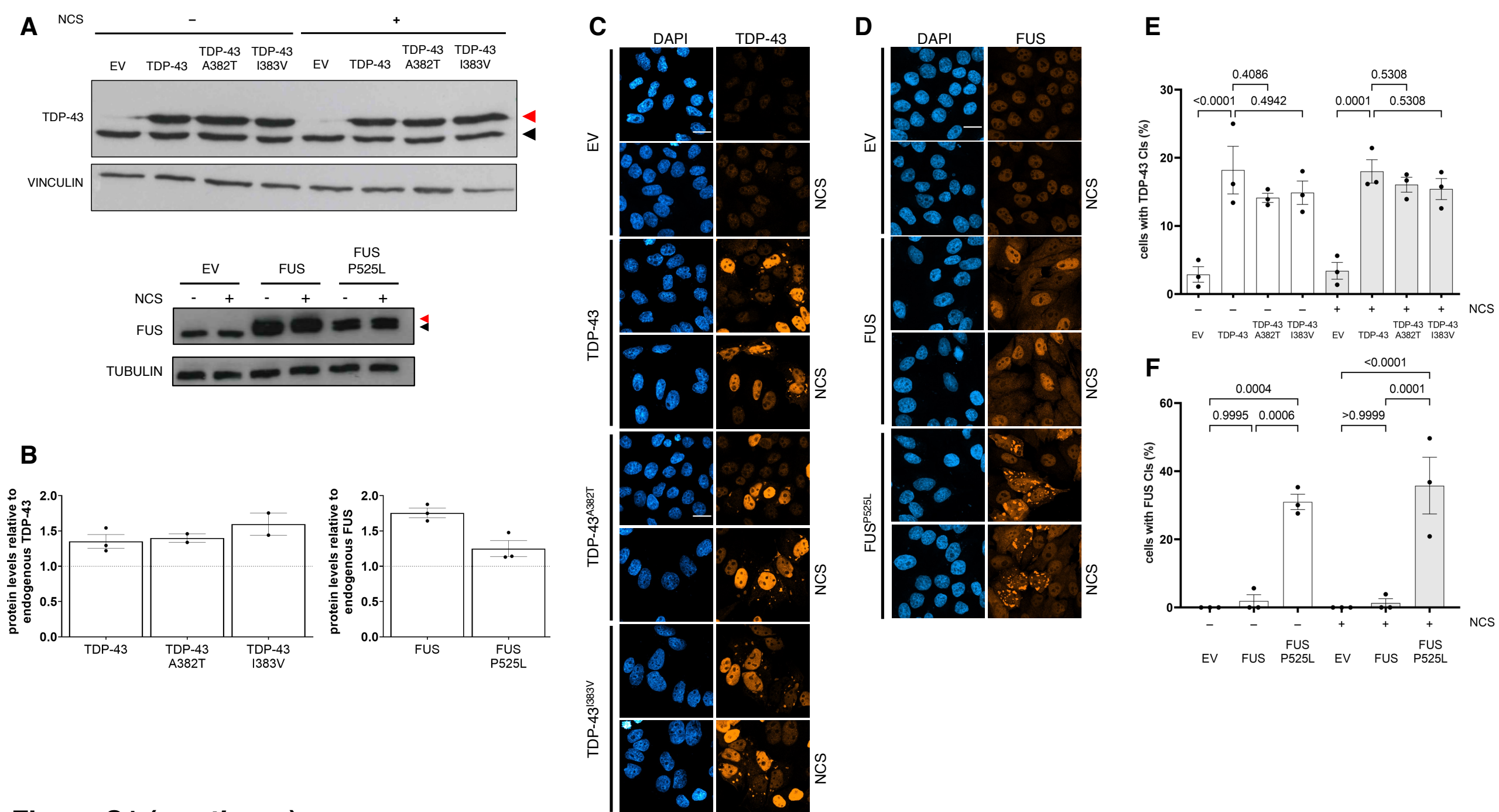

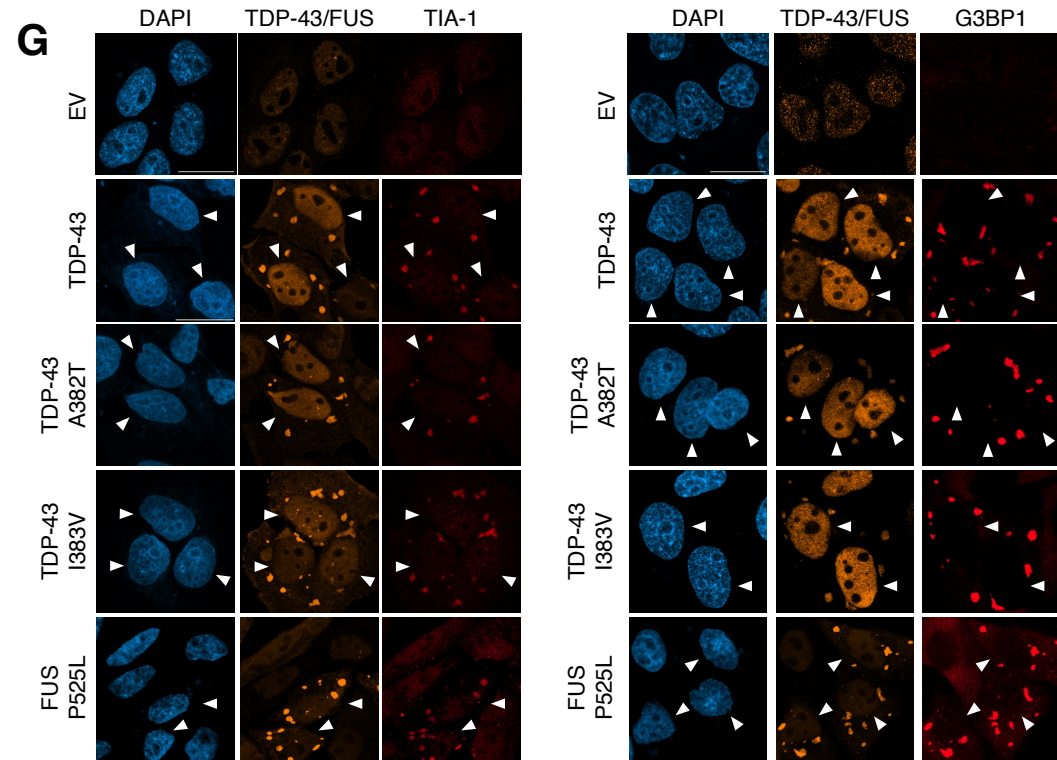

**H**

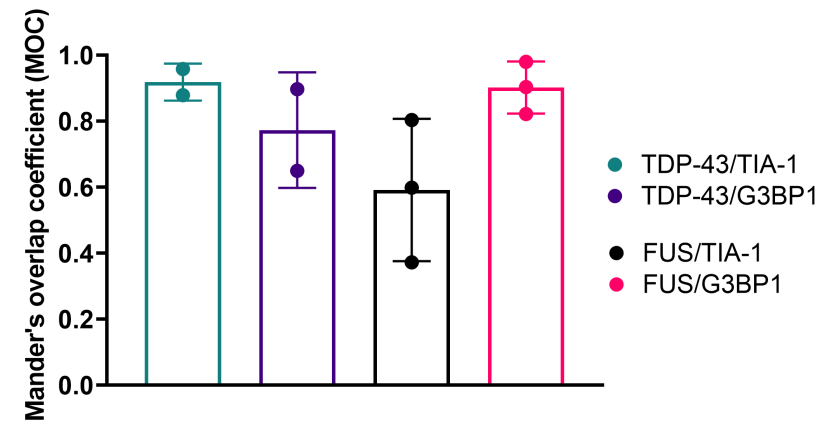

**Figure S1 (continued)**

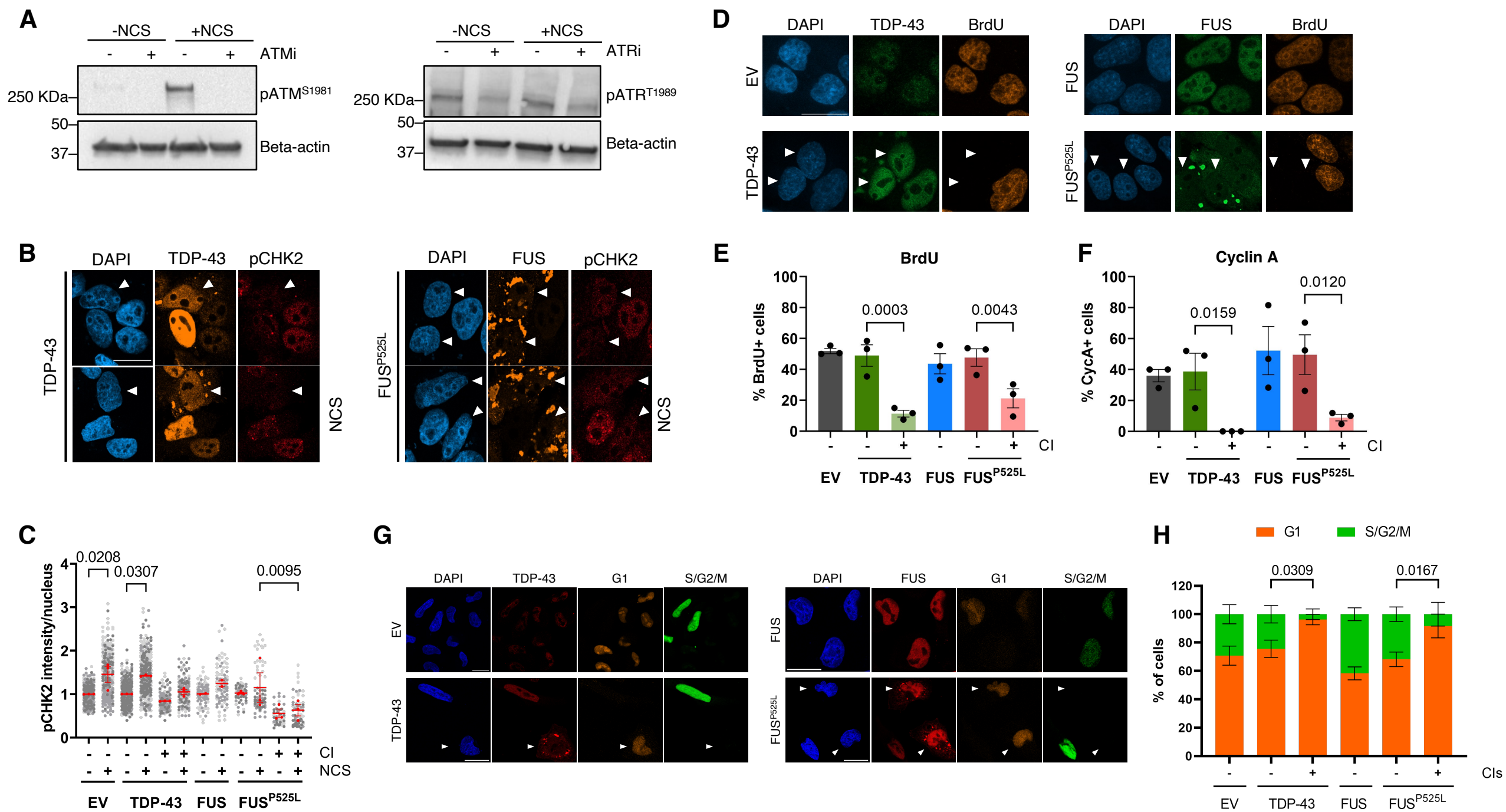

**Figure S2**

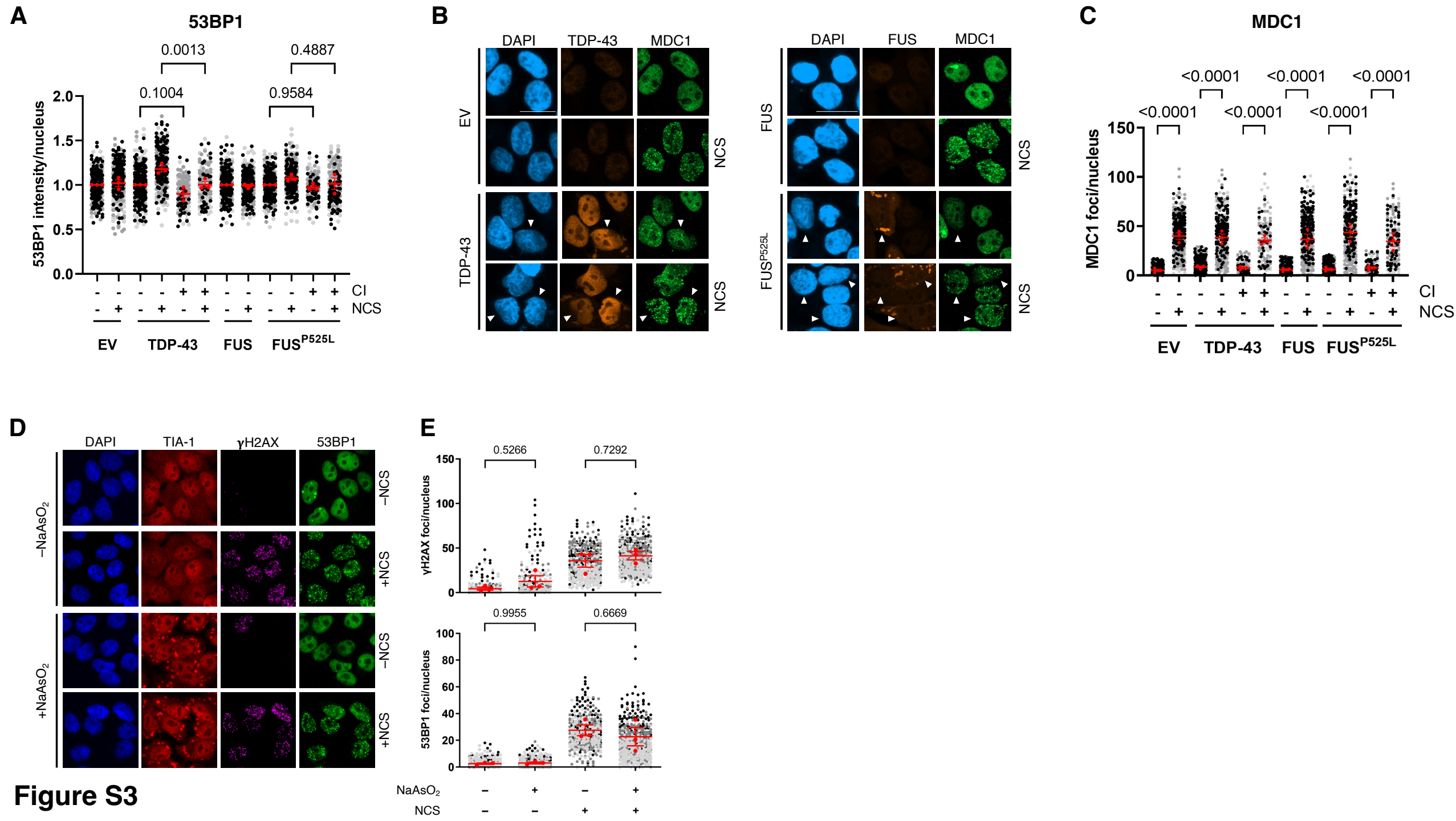

Figure S3

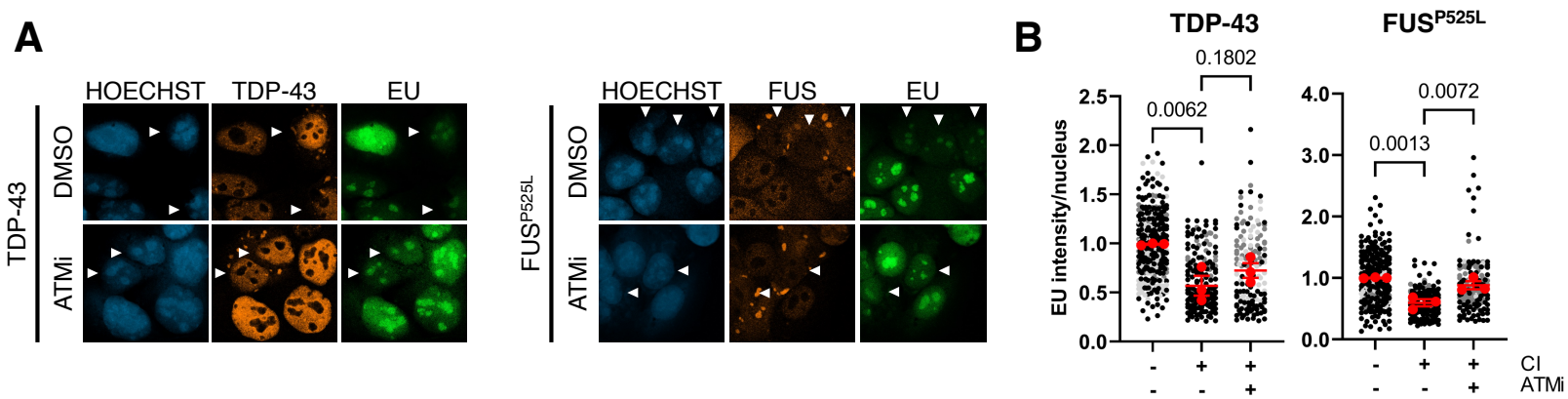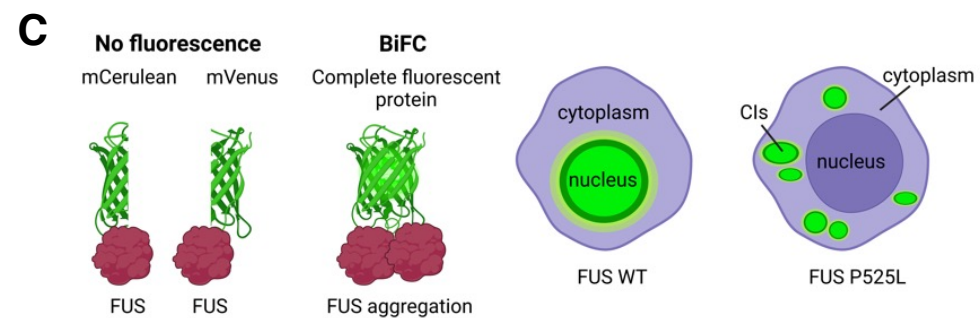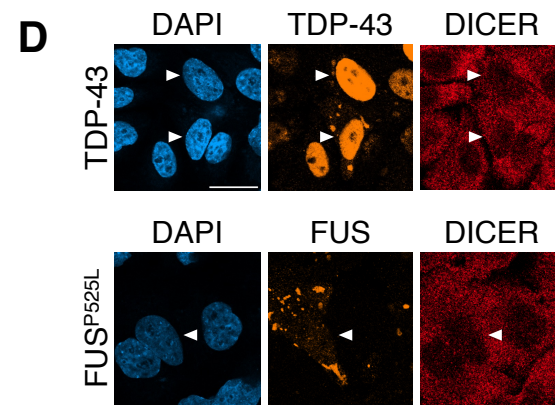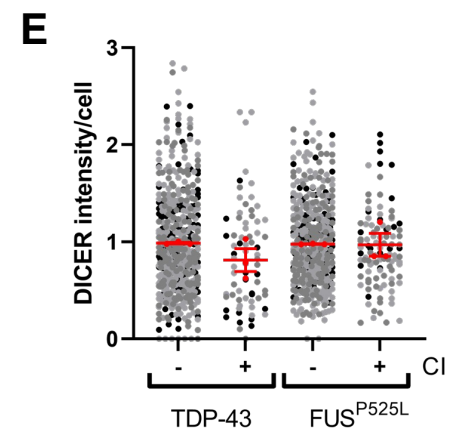

**Figure S4**

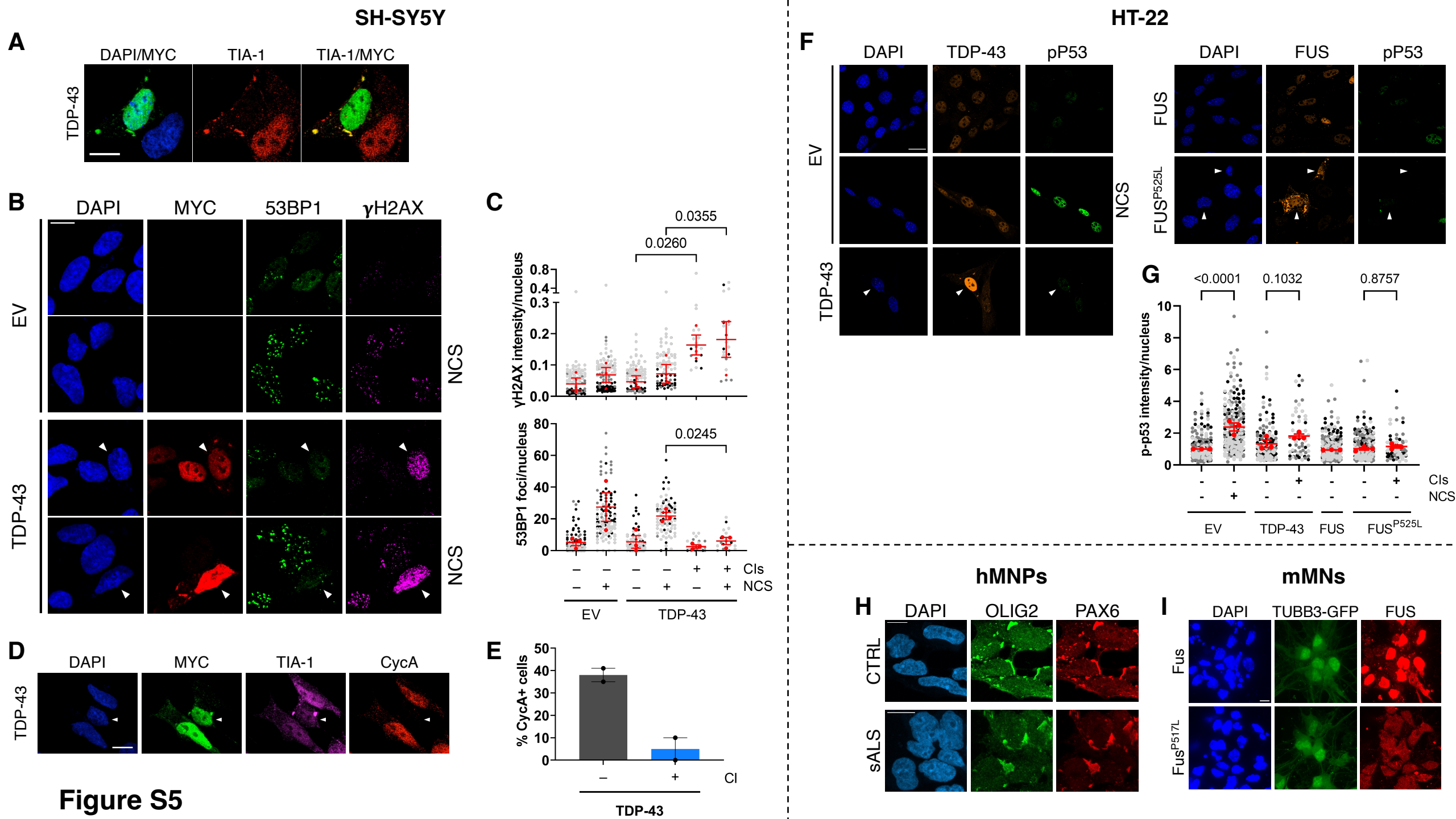

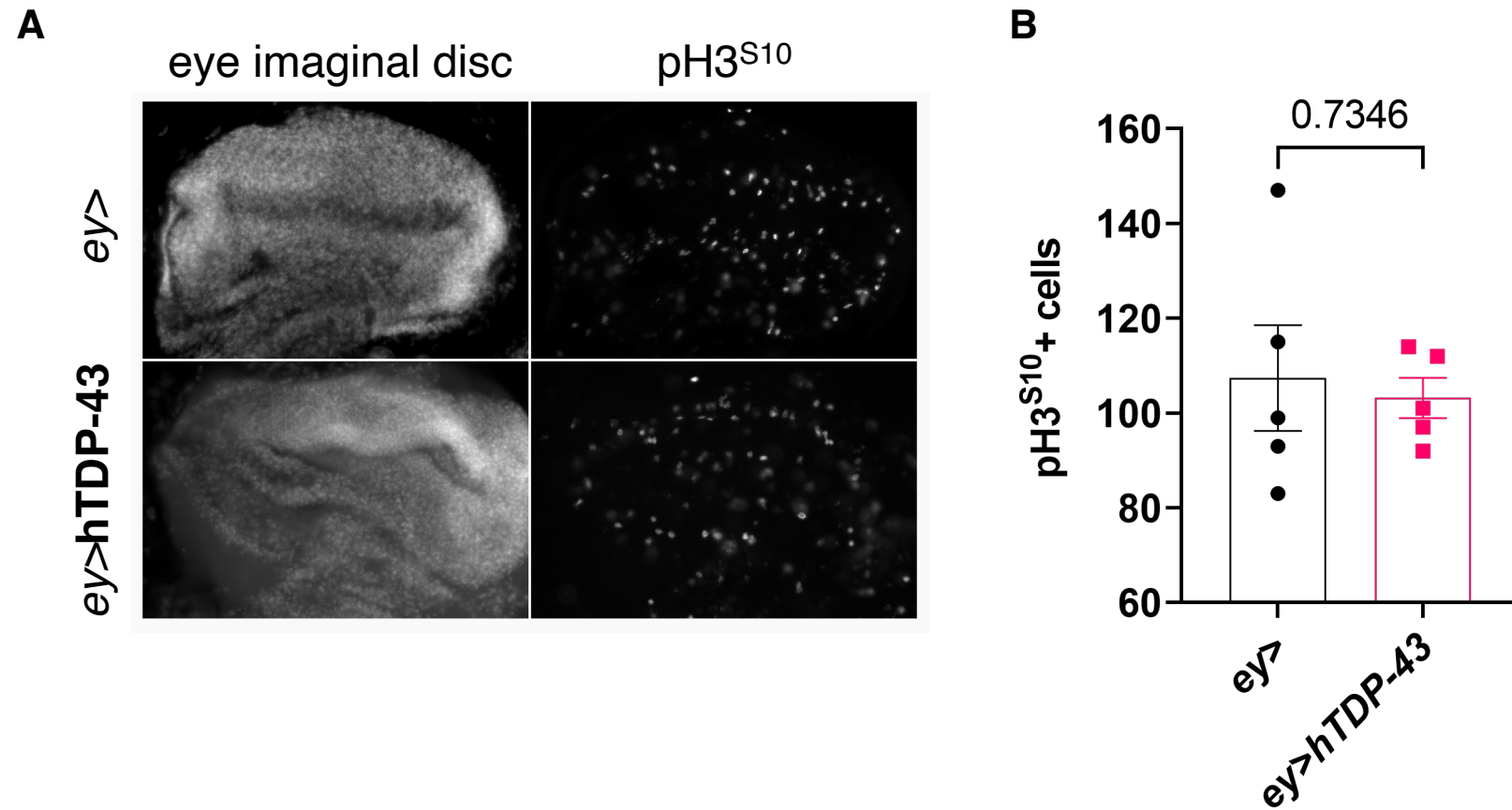

Figure S6 (related to Figure 7)

Supplement: Supplementary file 1 — Supplementary Figures 1-6 [file 41418_2025_1530_MOESM1_ESM.pdf]

# Related to Figure S1A

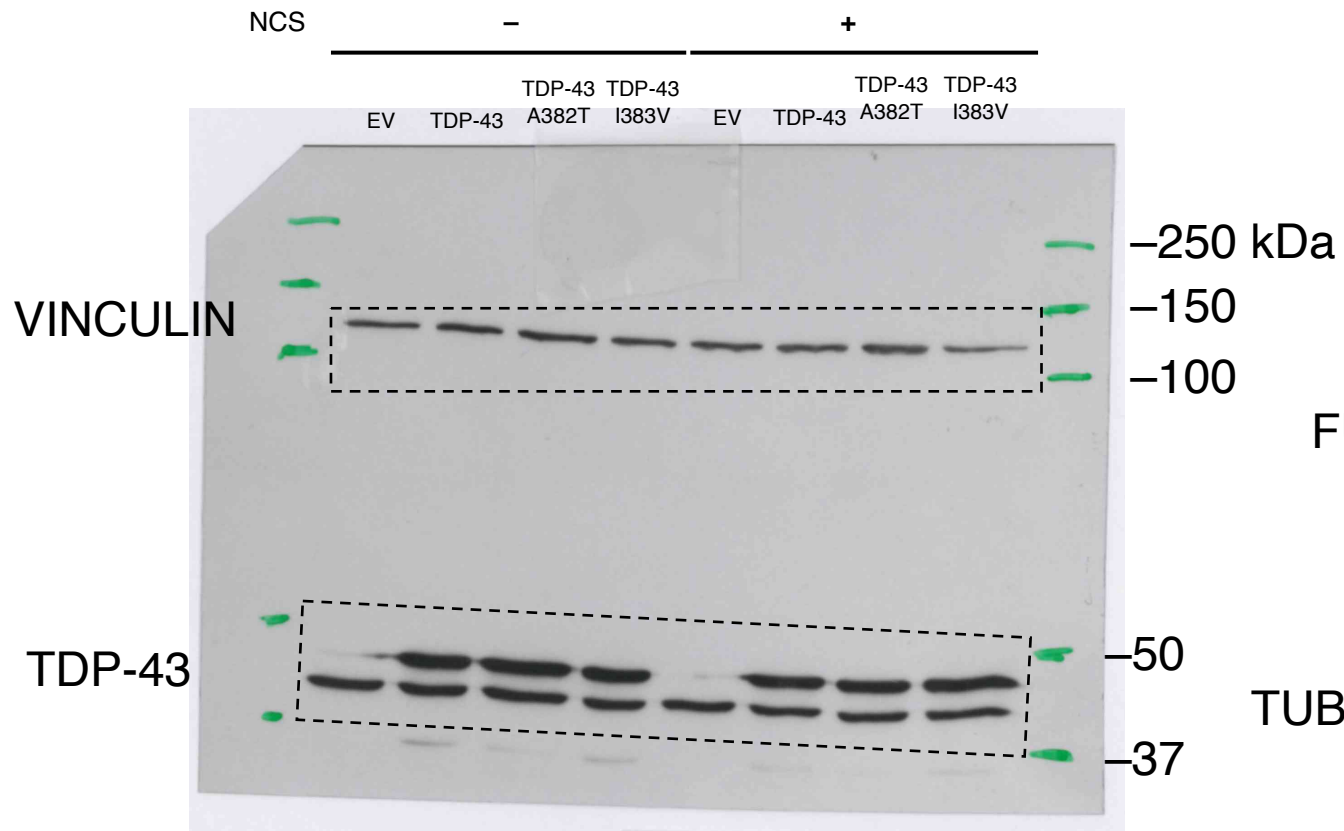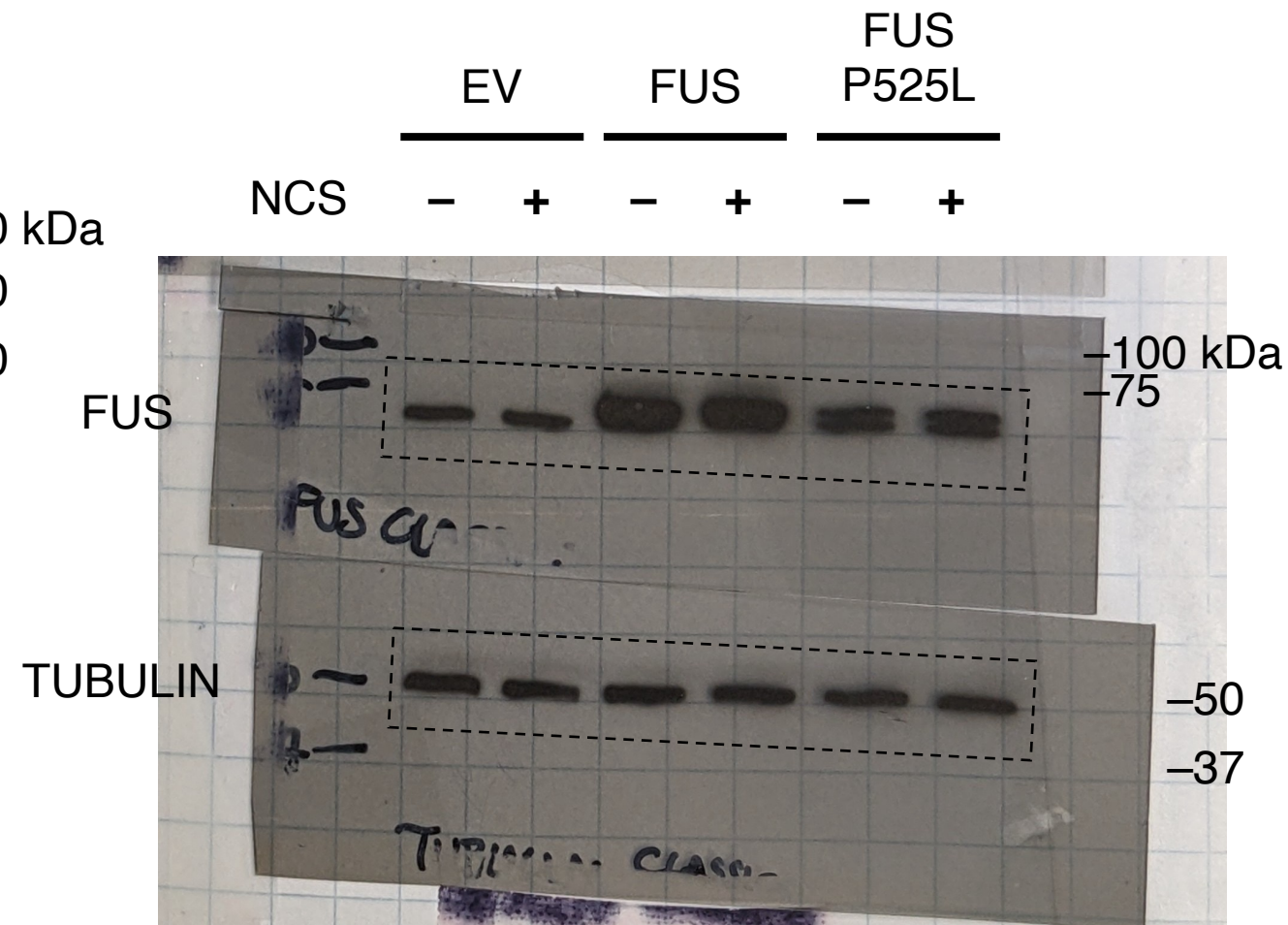

Related to **Figure S2A**

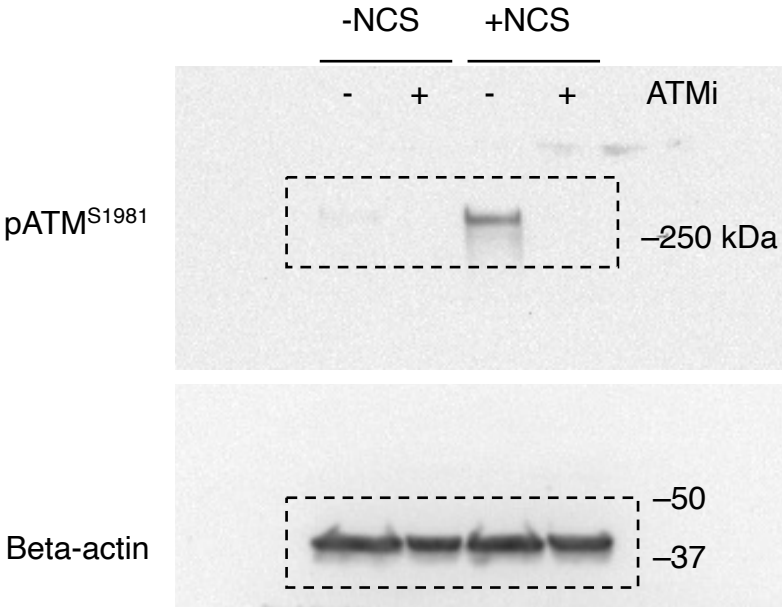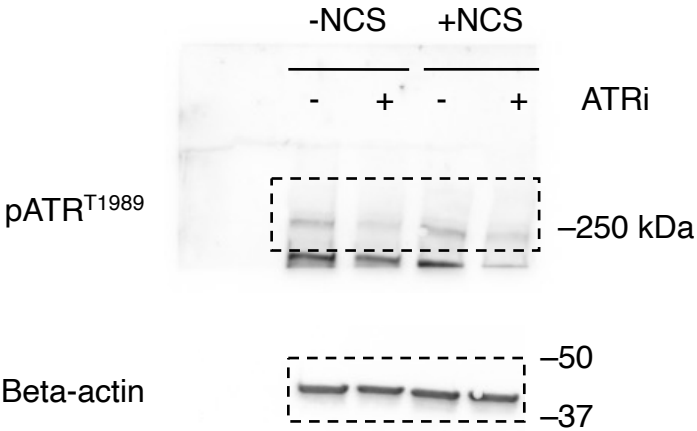

Supplement: Supplementary file 4 — Original data files [file 41418_2025_1530_MOESM4_ESM.pdf]
