# Supplementary material for: DNA damage response defects induced by the formation of TDP-43 and mutant FUS cytoplasmic inclusions and their pharmacological rescue
Source: Cell Death Differ. 2025 May 29;32(12):2309–22. doi: 10.1038/s41418-025-01530-7 (PMC12669588; doi:10.1038/s41418-025-01530-7)
Supplement: Supplementary file 2 — Supplementary Figure Legends [file 41418_2025_1530_MOESM2_ESM.docx]

**SUPPLEMENTARY FIGURE LEGENDS**

**Figure S1 (related to Figure 1). TDP-43 and FUS^P525L^ CIs colocalize with SGs. A)** Analysis by immunoblot of TDP-43 and FUS levels in damaged (NCS +) or undamaged (NCS -) HeLa cells transfected with the indicated plasmids (EV = empty vector); Vinculin and Tubulin were used as loading controls. Black and red arrowheads indicate endogenous and ectopically expressed proteins, respectively. **B)** The histograms show the ratio between the transfected TDP-43 and FUS protein levels and their endogenous protein amounts as determined in A; values are the means ± SEM of at least 2 independent experiments. **C, D)** Immunofluorescence (IF) analysis of endogenous TDP-43 and FUS subcellular localization in damaged (NCS) or undamaged HeLa cells transfected with plasmids expressing wild-type or mutant TDP-43 or FUS; cells transfected with EV were used as a control; nuclei were counter-stained with DAPI; scale bar = 10 µm. **E, F)** Histograms showing the percentage of HeLa cells harboring wild-type or mutant TDP-43 of FUS CIs. Values are the means ± SEM of three independent experiments. **G)** Analysis by IF of endogenous TDP-43 and FUS (orange) co-localizing with stress granules (SGs), labelled by TIA-1 or G3BP1 staining (red), in cells expressing wild-type, mutant TDP-43, FUS^P525L^, or transfected with EV as a control; nuclei were counter-stained with DAPI; arrowheads mark cells with CIs; scale bar = 20 µm. **H)** The histograms show the Mander’s Overlap Coefficient (MOC) between TDP-43 or FUS signals and those of the SG markers TIA-1 and G3BP1. Values are the means ± SEM of at least two independent experiments.

**Figure S2 (related to Figure 2). Cells with TDP-43 and FUS^P525L^ CIs arrest preferentially in G1. A)** Analysis by immunoblot of ATM and ATR activation (pATM^S1981^ and pATR^T1989^, respectively) in damaged (+NCS) or undamaged (-NCS) HeLa cells treated with ATMi or ATRi; cells treated with DMSO only (-) were used as a control. **B)** Representative images of pCHK2 signals (red) in damaged (NCS) or undamaged HeLa cells transfected with plasmids encoding for TDP-43 or FUS^P525L^; endogenous TDP-43 and FUS are labelled in orange; nuclei were counter-stained with DAPI; arrowheads mark cells with CIs; scale bar = 10 µm. **C)** Quantification of pCHK2 signals in cells with or without cytoplasmic inclusions (± CI) determined in B. The red dots of the super-plot represent the mean values of each biological replicate; red bars indicate the averages ± SEM of three independent experiments. **D)** Immunofluorescence (IF) images of BrdU incorporation assay in HeLa cells transfected with plasmids expressing TDP-43, FUS or FUS^P525L^; cells transfected with an empty vector (EV) were used as a control; endogenous TDP-43 and FUS are shown in green; nuclei were counter-stained with DAPI; arrowheads mark cells with CIs; scale bar = 10 µm. **E)** Histograms showing the percentage of BrdU-positive cells with or without cytoplasmic inclusions (± CI) determined in D; values are the means ± SEM of three independent experiments. **F)** The histograms show the percentage of cells, containing or not cytoplasmic inclusions (± CI), that express Cyclin A (CycA+); values are the means ± SEM of three independent experiments. **G)** Representative images of cell cycle profiles of FUCCI-expressing HeLa cells transfected with plasmids encoding for TDP-43, FUS or FUS^P525L^; samples transfected with EV are also examined. Cells in G1 (i.e., expressing CDT1) are shown in orange, while those in S/G2/M (i.e., expressing Geminin) are labelled in green; endogenous TDP-43 and FUS are also shown (red); nuclei are counter-stained with DAPI (blue); arrowheads mark cells with CIs; scale bar = 20 µm. **H)** Histograms showing the percentage of HeLa cells, with or without CIs, in G1 or in S/G2/M phases, as determined in G. Values are the means ± SEM of three independent experiments.

**Figure S3 (related to Figure 3). A)** Quantification of 53BP1 intensity in damaged (NCS +) and undamaged HeLa cells with or without cytoplasmic inclusions (± CI) determined in Figure 3A. The red dots of the super-plot represent the mean values of each biological replicate; red bars indicate the averages ± SEM of three independent experiments. **B)** Immunofluorescence (IF) analysis of MDC1 recruitment in damaged (NCS) or undamaged HeLa cells transfected with plasmids encoding for TDP-43, FUS or FUS^P525L^; cells transfected with an empty vector (EV) were used as a control; endogenous TDP-43 and FUS are labelled in orange; nuclei were counter-stained with DAPI; arrowheads mark cells with CIs; scale bar = 10 µm. **C)** Quantification of MDC1 foci in cells with or without cytoplasmic inclusions (± CI) determined in B. The red dots of the super-plots represent the mean values of each biological replicate; red bars indicate the averages ± SEM of three independent experiments. **D)** Analysis by IF of γH2AX (magenta) and 53BP1 (green) signals in damaged or undamaged (±NCS) HeLa cells treated or not with sodium arsenite (±NaAsO_2_); cells were also stained for the SG marker TIA-1 (red); nuclei were counter-stained with DAPI (blue). **E)** Quantification of γH2AX and 53BP1 foci in HeLa cells treated as in D. The red dots of the super-plot represent the mean values of each biological replicate; red bars indicate the averages ± SEM of three independent experiments.

**Figure S4 (related to Figure 4). A)** Representative images of HeLa cells expressing TDP-43 or FUS^P525L^, treated with ATMi or with the vehicle only (DMSO) and pulse-labelled with EU; endogenous TDP-43 and FUS are labelled in orange; nuclear DNA was visualized using Hoechst dye; arrowheads mark cells with CIs. **B)** Quantification of EU signals in cells with or without cytoplasmic inclusions (± CI) as determined in A. The red dots of the super-plots represent the mean values of each biological replicate; red bars indicate the averages ± SEM of three independent experiments. **C)** Scheme depicting the Bimolecular fluorescence complementation (BiFC) system used. **D)** Representative images of DICER protein levels (red) in HeLa cells expressing TDP-43 or FUS^P525L^; endogenous TDP-43 and FUS are shown in orange; nuclei were counter-stained with DAPI; arrowheads mark cells with CIs; scale bar = 20 µm. **E)** Quantification of DICER signals in cells with or without cytoplasmic inclusions (± CI) determined in D. The red dots of the super-plot represent the mean values of each biological replicate; red bars indicate the averages ± SEM of three independent experiments.

**Figure S5 (related to Figure 5). A)** Representative images of myc-tagged TDP-43 (green) co-localizing with TIA-1 (red) in SH-SY5Y cells transfected with the plasmid encoding for TDP-43; nuclei are counter-stained with DAPI (blue); scale bar = 10 µm. **B)** Analysis by immunofluorescence (IF) of γH2AX (magenta) and 53BP1 (green) signals in damaged (NCS) or undamaged SH-SY5Y cells expressing TDP-43; cells transfected with the empty vector (EV) are also examined; myc-tagged TDP-43 is shown in red; nuclei are counter-stained with DAPI (blue); arrowheads mark cells with CIs; scale bar = 10 µm. **C)** Quantification of γH2AX intensity and 53BP1 foci in SH-SY5Y cells with or without cytoplasmic inclusions (± CI) determined in B. The red dots of the super-plot represent the mean values of each biological replicate; red bars indicate the averages ± SEM of three independent experiments. **D)** IF images of TDP-43-expressing SH-SY5Y stained for Cyclin A (CycA, red) and TIA-1 (magenta); myc-tagged TDP-43 is labelled in green; nuclei are counter-stained with DAPI (blue); arrowheads mark cells with CIs; scale bar = 10 µm. **E)** Histograms showing the percentage of SH-SY5Y cells, containing or not cytoplasmic inclusions (± CI), that express Cyclin A (CycA+), determined in D; values are the means ± SEM of two independent experiments. **F)** Analysis by IF of p53 activation in HT-22 cells treated as in Figure 5A. Cells were stained for phospho-p53 (Ser 15) (p-p53, green) and for endogenous TDP-43/FUS (orange); nuclei were counter-stained with DAPI (blue); arrowheads mark cells with CIs; scale bar = 20 µm. **G)** Quantification of p-p53 intensity in HT-22 cells with or without cytoplasmic inclusions (± CI) determined in F. The red dots of the super-plot represent the mean values of each biological replicate; red bars indicate the averages ± SEM of three independent experiments. **H)** hMNPs derived from sALS or healthy individuals (CTRL) were stained for OLIG2 (green) and PAX6 (red) markers; nuclei were counter-stained with DAPI; scale bar = 10 µm. **I)** Representative images of TUBB3-GFP-expressing mMNs carrying wild-type or mutant Fus (labelled in red); nuclei were counter-stained with DAPI; scale bar = 100 µm.

**Figure S6 (related to Figure 7). A)** Immunofluorescence analysis of cell proliferation in eye imaginal discs of Drosophila larvae expressing or not hTDP-43 through staining for the mitotic marker phospho-H3 (Ser 10) (pH3^S10^). **B)** Quantification of the number of mitotic cells (pH3^S10^-positive) in each eye imaginal disc as determined in A. Values are the means ± SEM; five discs were analyzed for each genotype.
