# Supplementary material for: DNA damage response defects induced by the formation of TDP-43 and mutant FUS cytoplasmic inclusions and their pharmacological rescue
Source: Cell Death Differ. 2025 May 29;32(12):2309–22. doi: 10.1038/s41418-025-01530-7 (PMC12669588; doi:10.1038/s41418-025-01530-7)
Supplement: Supplementary file 3 — Supplementary Tables [file 41418_2025_1530_MOESM3_ESM.docx]

**Supplementary Table 1: antibodies used in this study.**

| **Target** | **Host** | **Dilution** | **Application** | **Manufacturer** | **Cat. N.** |
| --- | --- | --- | --- | --- | --- |
| Primary Antibodies | | | | | |
| TDP-43 | goat | 1: 200 | IF | Arigo | ARG64788 |
| TDP-43 | rabbit | 1:500 | IF | Proteintech | 10782-2-AP |
| FUS | goat | 1:1000 | IF | Bethyl | A303-839A |
| FUS | rabbit | 1:1000 | IF | Bethyl | A300-293A |
| γH2AX | mouse | 1:1000 | IF | Millipore | 05-636 |
| γH2AX | rabbit | 1:1000 | IF (mMNs) | Abcam | Ab11174 |
| Anti-Beta III Tubulin | chicken | 1:200 | IF | Sigma-Aldrich | AB9354 |
| TDP-43 | rabbit | 1:1000 | WB | Proteintech | 10782-2-AP |
| FUS | mouse | 1:1000 | WB | Santa Cruz | sc-47711 |
| VINCULIN | mouse | 1:2000 | WB | Millipore | MAB3574 |
| TIA-1 | rabbit | 1:200 | IF | Abcam | ab40693 |
| G3BP1 | mouse | 1:100 | IF | BD Laboartories | 611126 |
| pATM (pSer1981) | mouse | 1:600 | IF | Rockland | 200-301-400 |
| pATM (pSer1981) | mouse | 1:2000 | WB | Millipore | 05-740-25UG |
| pATR (pThr1989) | rabbit | 1:200 | WB | Abcam | Ab227851 |
| p-p53-Ser15 | rabbit | 1:400 | IF | Cell Signaling | 9284 |
| β-Actin | mouse | 1:800 | WB | Santa Cruz | Sc-58673 |
| pCHK2 | rabbit | 1:100 | IF | Novus | NB100-92502 |
| Cyclin A | mouse | 1:200 | IF | Santa Cruz | sc271-682 |
| 53BP1 | goat | 1:1000 | IF | Bethyl | A303-906A |
| p53BP1 | rabbit | 1:400 | IF | Cell Signaling | 2675 |
| MDC1 | mouse | 1:500 | IF | Sigma | M2444 |
| DROSHA | rabbit | 1:500 | IF | Abcam | ab183732 |
| DROSHA | rabbit | 1:500 | IF (mMNs) | Cell Signaling | 3364 |
| DICER | mouse | 1:100 | IF | Abcam | ab14601 |
| γH2AX | rabbit | 1:1000 | IHC | Abcam | ab11174 |
| 53BP1 | rabbit | 1:1000 | IHC | Novus | NB100-304 |
| BrdU | mouse | 1:20 | IF | BD Laboratories | 347580 |
| phospho-H3-Ser10 |  | 1:500 | IF | Merck |  |
| Secondary Antibodies | | | | | |
| Anti-mouse IgG HRP | donkey | 1:10 000 | WB | Abcam | ab97030 |
| Anti-rabbit IgG HRP | donkey | 1:10 000 | WB | Abcam | ab97064 |
| Anti-goat  Alexa 488  IgG | donkey | 1:400 | IF | Abcam | ab150129 |
| anti-mouse  Alexa 647  IgG | donkey | 1:400 | IF | Abcam | ab150107 |
| Anti-rabbit  Alexa 488  IgG | donkey | 1:400 | IF | Abcam | ab150073 |
| anti-rabbit  Alexa 647  IgG | donkey | 1:400 | IF | Abcam | ab150075 |
| Anti-mouse  Alexa 488  IgG | donkey | 1:400 | IF | Abcam | ab150105 |
| anti-goat  Alexa 647  IgG | donkey | 1:400 | IF | Abcam | ab150131 |
| anti-rabbit  Alexa 555  IgG | donkey | 1:400 | IF | Invitrogen | A31572 |
| anti-mouse  Alexa 555  IgG | donkey | 1:400 | IF | Invitrogen | A31570 |
| anti-goat  Alexa 555  IgG | donkey | 1:400 | IF | Invitrogen | A21432 |
| anti-chicken  Alexa 488A  IgG | donkey | 1:200 | IF (mMNs) | Sigma-Aldrich | SAB4600031 |
| anti-rabbit Oregon Green | donkey | 1:300 | IF (Drosophila) | Jackson Immunoresearch |  |

**Supplementary Table 2: primers used in this study.**

| **Gene** | **5’-3’ sequence** | **Application** |
| --- | --- | --- |
| DROSHA_FW | GGCCCGAGAGCCTTTTATAG | Standard RT-qPCR |
| DROSHA REV | TGCACACGTCTAACTCTTCCAC | Standard RT-qPCR |
| RPLP0_FW | ATGCCCAGGGAAGACAGGGCG | Standard RT-qPCR |
| RPLP0_REV | CGAAGGGACATGCGGATCTGCTGC | Standard RT-qPCR  Strand specific RT |
| LAC_from_F1 | GGTCACCGGTTCTACATGTAGCCACACATTGTTATCCG | dilncRNA detection |
| LAC_from_R1 | CGTGGCAATCTGTGGCCTCATGTGGACTTGTGAGTG | dilncRNA detection Strand specific RT |
